# Incorporation of genome-bound cellular proteins into HIV-1 particles regulates viral infection

**DOI:** 10.1016/j.celrep.2026.117090

**Published:** 2026-04-07

**Authors:** Manuel Garcia-Moreno, Azman Embarc-Buh, Robin Truman, Marko Noerenberg, Louisa Iselin, Honglin Chen, Caroline E. Lenz, Jeffrey Y. Lee, Kate Dicker, Snehith Dyavari Shetty, Natasha Palmalux, Quan Gu, Thibault J.M. Sohier, Aino I. Järvelin, Wael Kamel, Vincenzo Ruscica, Emiliano P. Ricci, Ilan Davis, Shabaz Mohammed, Alfredo Castello

**Affiliations:** 1 Department of Biochemistry, University of Oxford, Oxford OX1 3QU, UK; 2 MRC-University of Glasgow Centre for Virus Research, Glasgow G61 1QH, UK; 3 Nuffield Department of Medicine, University of Oxford, Oxford OX1 3SY, UK; 4 Dunn School of Pathology, University of Oxford, Oxford OX1 3RE, UK; 5 School of Molecular Biosciences, College of Medical, Veterinary & Life Science, University of Glasgow, Wolfson Link Building, University Avenue, Glasgow G12 8QQ, UK; 6 Laboratoire de Biologie et Modelisation de la Cellule, Ecole Normale Superieure de Lyon, CNRS UMR 5239, Inserm U1293, UCBL, 46 allee d’Italie 69364 Lyon, France; 7 The Rosalind Franklin Institute, Harwell Campus, Didcot OX11 0FA, UK

**Keywords:** HIV, RNA, RNA-binding protein, virus, virion, viral particle, Rev, RNA-interactome capture, PURA

## Abstract

The initial steps of the human immunodeficiency virus type 1 (HIV-1) life cycle are regulated by cellular RNA-binding proteins, but only a few have been identified. Here, we developed *in virion* RNA interactome capture (ivRIC) to comprehensively profile the direct protein interactors of the HIV-1 genomic (g)RNA inside the viral particles. We identified 104 cellular RNA-binding proteins in virions (ivRBPs), many of which are nuclear. We determined the interactome of the viral RBP Rev and discovered that nuclear ivRBPs may associate gRNA in the nucleus and continue bound after the genesis of the viral particles. We also observed that ivRBPs are not incorporated into viral particles based on their abundance, but likely through selective mechanisms. Moreover, we show that the ivRBPs PURA and its homolog PURB control HIV-1 particle infectivity and engage with several viral proteins and key elements within HIV-1 gRNA, showcasing the importance of ivRBPs for HIV-1 infection.

## Highlights

• ivRIC elucidates the composition of viral ribonucleoproteins inside virions
• 104 cellular ivRBPs bind to HIV-1 genomic (g)RNA within virions
• ivRBPs are selectively incorporated into virions
• PURA interacts with HIV-1 gRNA and promotes reverse transcription

## Graphical abstract

**Figure:**
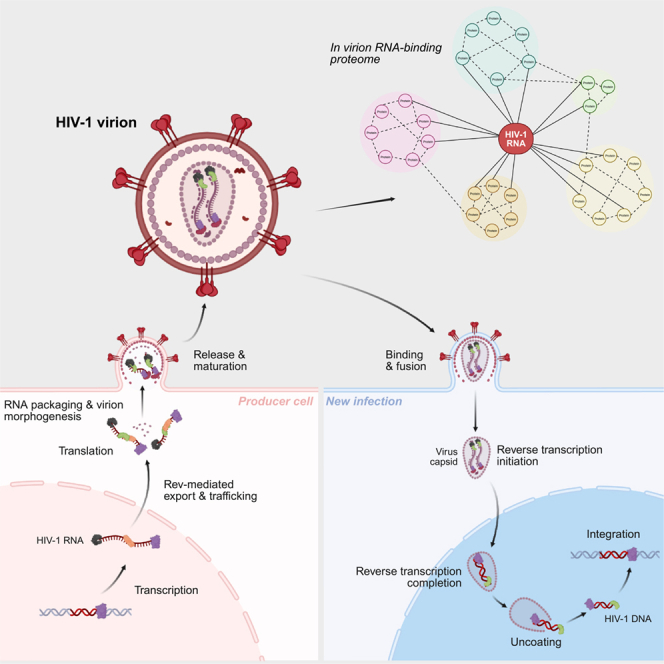

## Teaser

Does HIV-1 package host proteins that promote infection? Using *in virion* RNA interactome capture, Garcia-Moreno et al. mapped human proteins bound to the viral genome inside virions. They identified over 100 host proteins that are likely packaged selectively. One of them, PURA, supports reverse transcription and enables efficient early infection.

## Introduction

HIV-1 genomic (g)RNA is reverse transcribed into DNA and integrated into the host chromosome. Cellular RNA-binding proteins (RBPs) have been extensively connected to the following steps of the viral life cycle, which starts with the transcription of viral RNAs by the host RNA polymerase II and its co-factors and continues with their capping, splicing, and polyadenylation.^1^ HIV-1 produces several viral RNA species that can be divided into single spliced, fully spliced, and unspliced (i.e., genome) RNAs.^2^ Fully spliced HIV-1 RNAs are transported to the cytoplasm using the canonical mRNA export pathways.^3^ However, unspliced and partially spliced RNAs must interact with the viral RBP Rev to hijack CRM1 and avoid nuclear retention.^2^^,^^4^ Viral RNA stability and decay, translation, and RNA transport also involve cellular RBPs and complexes, including the ribosome.^1^^,^^2^^,^^5^^,^^6^^,^^7^^,^^8^^,^^9^^,^^10^ Recent reports have suggested roles of cellular RBPs in viral particle formation and posterior steps such as reverse transcription and integration. However, the scope and relevance of these host-virus interactions remain poorly characterized.^9^

It was thought that HIV-1 particles disassemble upon cell entry, releasing their (g)RNA molecules into the cytoplasm. However, recent paradigm-shifting advances challenged this view, showing that the capsid core can remain intact during its transit throughout the cell and can be visualized in the nuclear pore and nucleoplasm.^11^^,^^12^^,^^13^^,^^14^ Moreover, it is now accepted that reverse transcription can occur inside the capsid core.^11^^,^^15^^,^^16^ Several cellular RBPs have been proposed to regulate HIV-1 particle assembly and reverse transcription.^9^ However, the capsid-confined reverse transcription model implies that host proteins involved in this process must be present inside the virion. Proteomic analyses of purified HIV-1 particles revealed the presence of hundreds of RBPs within virions.^9^^,^^17^^,^^18^^,^^19^^,^^20^^,^^21^ While exciting, these proteomic analyses were affected by biological and technical limitations, including (1) the uptake of a portion of the cytosol by budding particles, leading to the passive acquisition of bystander proteins; (2) the presence of extracellular vesicles with similar sizes to virions; and (3) the lack of appropriate negative controls and/or quantitative information.^17^^,^^18^^,^^19^^,^^20^^,^^21^ Therefore, progress in elucidating the scope of host RBPs incorporated into virions requires the development of new strategies to differentiate between passive bystanders and proteins actively engaging with the gRNA.

Here, we applied a new approach to determine the complement of proteins that interact with the HIV-1 gRNA inside the viral particles called *in virion* RNA interactome capture (ivRIC). We discover that the *in virion* packaged genomic ribonucleoprotein (ivRNP) contains over 100 cellular proteins, many of which are nuclear. We show that many of these nuclear RBPs are also interactors of the viral RBP Rev, suggesting that they associate with the viral RNP during its nuclear life. Moreover, we found that the components of the ivRNPs play important regulatory roles in HIV-1 infection. Particularly, PURA and PURB bind to critical regulatory elements on the HIV-1 gRNA and the viral reverse transcriptase (RT), aiding viral particle infectivity probably through the regulation of reverse transcription. Our study thus provides a new landscape of host-HIV interactions with regulatory potential.

## Results

### ivRIC, a new approach to analyze the composition of the viral RNPs assembled into virions

**Figure 1:**
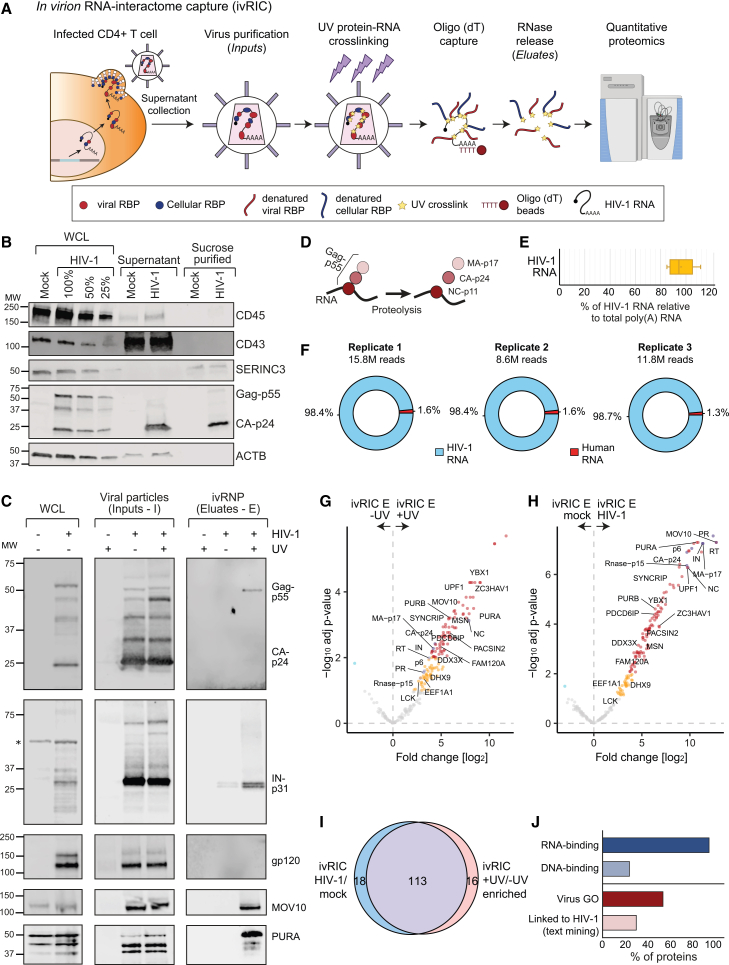
*In virion* RNA interactome capture (ivRIC) reveals the composition of the genomic ribonucleoprotein packaged within HIV-1 virions (ivRNP) (A) Schematic representation of the ivRIC protocol. Samples and controls are depicted in Figure S1. (B) Western blotting of the viral particles before and after sucrose cushion purification using antibodies against markers of extracellular vesicles (CD45, CD43, and SERINC3) and viral proteins; representative gel of *n* = 3. (C) Western blotting of the whole-cell lysates (WCLs), inputs, and eluates of a representative ivRIC experiment performed in CD4^+^ T-lymphocytic (SupT1) cells. Asterisk marks unspecific bands; representative gel of *n* = 3. (D) Schematic representation of the proteolysis of the Gag polyprotein. *MA*, matrix; *CA*, capsid; *NC*, nucleocapsid. (E) Relative proportion of the HIV-1 gRNA in ivRIC eluates estimated by absolute RT-qPCR; *n* = 3; the line within the box represents the mean and error bars ± standard deviation (SD). (F) Analysis of oligo(dT) isolated RNA from sucrose purified HIV-1_mCherry-Nef_ particles by RNA-sequencing; *n* = 3. (G and H) Volcano plots of the quantitative proteomic analysis of the ivRIC eluates from virions produced by SupT1 cells; *n* = 4. Red and dark blue dots are proteins enriched with 1% FDR, while orange and cyan dots are proteins enriched with 10% FDR. Gray dots are non-enriched proteins. *E*, eluate. (I) Venn diagram showing the overlapping between the UV irradiated vs. non-irradiated samples and HIV-1-infected versus mock-infected comparisons. (J) Bar plots showing the proportion of ivRBPs in the ivRNP annotated with RNA- and DNA-binding (GO terms); virus-related (GO terms), and HIV-1-related (text-mining) functions. Related to Figure S1 and S2.

Proteomic analysis of purified viral particles revealed that hundreds of cellular proteins are incorporated into virions, including cellular RBPs.^9^ However, we noticed sparse overlapping between datasets (Figure S1A).^17^^,^^18^^,^^19^^,^^20^^,^^21^ This limited consistency is probably due to the impossibility to discriminate between bystander proteins captured passively during virion assembly and RBPs actively interacting with the HIV-1 gRNA. To identify RBPs directly bound to gRNA in HIV-1 particles, we developed ivRIC. In brief, viral particles are purified in a sucrose cushion, followed by protein-RNA “zero distance” ultraviolet (UV) cross-linking, lysis under denaturing conditions, and isolation of the polyadenylated HIV-1 gRNA with oligo(dT) magnetic beads (Figure 1A). RBPs are released by RNase treatment and identified by quantitative proteomics. ivRIC was applied to infectious HIV-1_mCherry-Nef_ particles purified from the supernatant of transfected HEK293T (Figures S1B, S1D, and S1E) or infected CD4^+^ T lymphocytic cells (SupT1; Figures 1B and S1C). The purification of viral particles from the supernatant of CD4^+^ T lymphocytic cells yielded strong enrichment of the viral capsid (CA-p24) relative to the whole-cell proteome (Figure 1B). In contrast, abundant cellular proteins such as albumin and β-actin were strongly depleted (Figures 1B and S1E). Moreover, extracellular vesicle markers (CD45, CD43 [also known as SPN], and SERINC3) were absent or markedly reduced compared with whole-cell lysates (Figure S1B). Trace SERINC3 signal was detected in purified samples but at comparable levels in supernatants from mock- and HIV-1-infected cells; we therefore included mock purifications to control for any potential carryover (Figures 1B and S1F).

Despite its high abundance in virions (∼2k copies/virion) and proximity to the viral RNA, the processed HIV-1 capsid (CA-p24) is depleted in eluates of the oligo(dT) capture, while the polyprotein Gag-p55 is enriched in a UV-dependent manner (Figures 1C and S1D). These striking results agree with our knowledge of the HIV-1 particle, where the CA-p24 forms a shell around the viral RNP, while the nucleocapsid (NC-p11) interacts with the viral RNA. CA-p24 can associate with viral RNA in immature particles as part of the polyprotein Gag-p55 because of the RNA-binding activity of NC-p11 (Figure 1D). In addition, the integrase (IN-p31) was also enriched in ivRIC eluates (Figure 1C), aligning with its reported RNA-binding activity.^22^ Conversely, the glycoprotein gp120 was absent in eluates, consistently with its location in the viral envelope (Figure 1C).

The success of ivRIC depends on the specificity and efficiency by which HIV-1 gRNA is isolated. Notably, HIV-1 gRNA represented ∼98% of the RNA detected in eluates (Figures 1E and 1F), implying that it is the major (if not the sole) contributor to the following proteomic results. These results were obtained by two orthogonal methods (absolute quantitative reverse transcription PCR [RT-qPCR] quantification and RNA sequencing) and are in agreement with studies corroborating that the population of cellular mRNA in viral particles is very small.^23^^,^^24^^,^^25^ Our RNA analysis also supports the efficiency of our purification protocol at removing potential sources of contamination such as extracellular vesicles and cellular debris.

**Figure 2:**
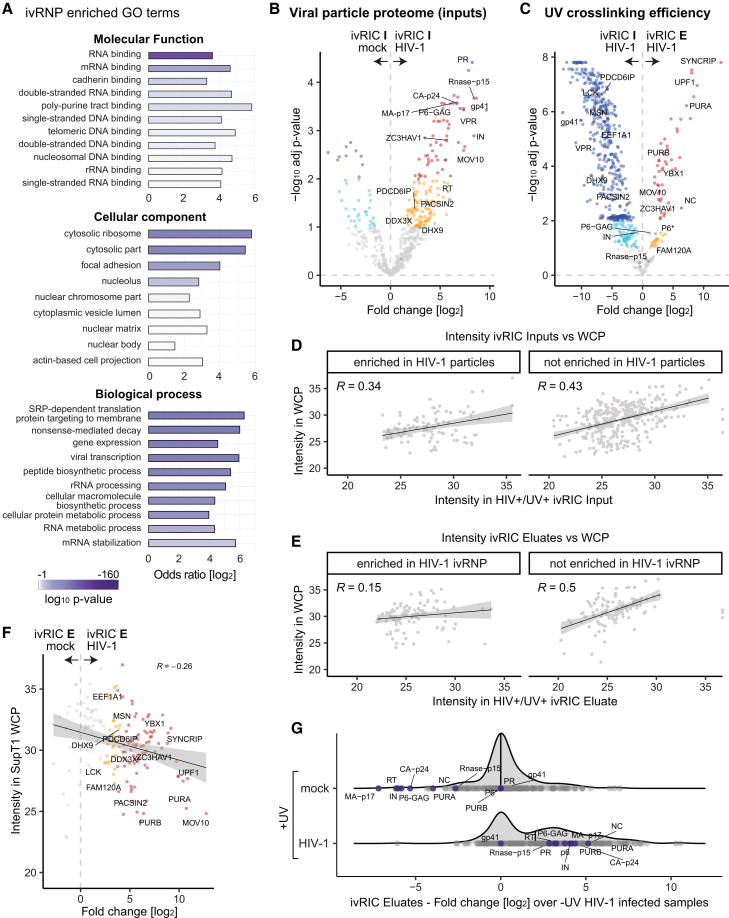
Analysis of the properties of ivRBPs (A) Analysis of the GO terms enriched in the ivRNP over the cellular proteome. (B and C) Volcano plots of the comparison between ivRIC inputs (I, purified viral particles) from HIV-1 infected and mock-infected cells (B) and ivRIC inputs versus eluates (E, ivRNP) (C); *n* = 4. Red and dark blue dots are proteins enriched with 1% FDR, while orange and cyan dots are proteins enriched with 10% FDR. Gray dots are non-enriched proteins. (D and E) Scatterplots showing the intensity of proteins in ivRIC inputs (D) or eluates (E) against the whole-cell proteome (WCP). (F) Scatterplot showing the protein intensity in the WCP (*y* axis) and the Log2 fold change in the ivRIC eluates from mock- and HIV-1-infected samples. Color code as in (B) and (C). (G) Density plots showing the distribution of fold changes in UV irradiated infected and non-infected samples over non-irradiated HIV-1-infected samples. Cellular proteins and viral proteins are shown in gray and purple, respectively. Related to Figure S3.

To identify the complement of cellular RBPs bound to the gRNA in viral particles, we analyzed the particles collected from CD4^+^ T cells by ivRIC in conjunction with quantitative proteomics (Figures S1F, S1G, and S2A). A total of 147 proteins were significantly enriched in eluates from UV-irradiated HIV-1-infected samples compared with either non-irradiated or mock-infected controls (Figures 1G–1I). Of these, 104 cellular and 9 viral proteins were consistently enriched relative to both controls (Figures 1G–1I; Table S1), which we designate as in-virion RNA-binding proteins (ivRBPs). We next validated our results by western blot, showing that the previously established ivRBP MOV10^26^^,^^27^ was strongly enriched in ivRIC eluates in a UV and infection-dependent manner (Figure 1C). Additionally, the newly discovered ivRBPs PURA and SUB1 were also detected in ivRIC eluates by western blotting (Figures 1C and S2B). As anticipated from a bona fide set of RBPs, most proteins enriched in ivRIC eluates were annotated with the “RNA binding” GO term and functions associated with RNA metabolism (Figures 1J, 2A, and S2). Interestingly, ∼25% of the ivRBPs also contain DNA-binding activity (Figures 1J and 2A), which is potentially significant given the RNA/DNA duality of HIV-1. Over half of the discovered ivRBPs are annotated with virus-related GO terms, and ∼25% of them are also linked to HIV-1 (Figure 1J), aligning with roles in infection. Notably, eight ivRBPs are involved in HIV-1 particle formation (PACSIN2, LCK, and PDCD6IP [also ALIX])^28^^,^^29^^,^^30^ or infectivity (MSN, UPF1, MOV10, DHX9, and EEF1A).^26^^,^^27^^,^^31^^,^^32^^,^^33^^,^^34^^,^^35^ Our data thus revealed that the viral genome engages with over 100 cellular RBPs within virions.

### ivRBPs are incorporated selectively into viral particles

Our next goal was to determine whether ivRBPs are incorporated into HIV-1 particles passively, based on their abundance, or selectively. For this, it was critical to characterize the proteome of the producer cells and purified particles (i.e., inputs of the ivRIC experiment). We identified 187 proteins enriched in the HIV-1-infected input samples over the mock controls, representing the viral particle proteome (Figures 2B, S3A, and S3B; Table S2). This dataset was additionally enriched in GO terms and pathways related to the plasma membrane and immunological receptors when compared to the ivRNP, reflecting the presence of plasma membrane-derived envelope in full viral particles (Figures S3C–S3E). We next calculated the protein intensity ratios between the inputs (total viral particle) and eluates (ivRNP), revealing two groups of proteins (Figure 2C). ivRBPs with high eluate/input ratios reflecting high cross-link-ability, typically supported by geometrically optimal protein-RNA interfaces and the presence of UV-favored amino acids and nucleotides. Additionally, ivRBPs with low ratios likely reflect transient, low-occupancy interactions (low bound/unbound) and/or suboptimal amino acid/nucleotide composition and spatial arrangement.

To discriminate between abundance-driven passive incorporation and selective packaging of proteins into virions, we normalized the protein intensities in ivRIC input (viral particle proteome) and eluates (ivRNP) against those in the whole CD4^+^ T cell proteome (WCP). Interestingly, the intensity of proteins in viral particles correlated well with protein abundance (R = 0.34; Figure 2D), while the ivRNP did so to a lower extent (R = 0.15; Figure 2E). When considering fold changes instead of raw intensities, the result was even more stark, with ivRBPs displaying anticorrelation with the WCP (Figure 2F and S2D).

A remaining question is whether ivRBPs are present in biologically meaningful quantities within virions. To test this, we compared the protein intensity and fold change distribution in eluates of ivRIC from HIV-1-infected and mock samples against the non-irradiated samples (i.e., no RNA-dependent purification).Viral proteins are present in both comparisons because they are detected, even if with low intensity, in the non-irradiated HIV-1-infected samples (Figure 2G). Strikingly, many cellular ivRBPs exhibited similar intensity levels and fold changes to HIV-1 proteins, which have high stoichiometry in viral particles (Figure 2G, gray vs. purple). These proteins however, had very small fold changes in the comparison to mock samples, suggesting that HIV-1 infection is an essential determinant of their high abundance in the RNPs isolated from the culture media. Several known regulators of HIV-1 particle infectivity were among the most prevalent ivRBPs in the gRNP, including UPF1^34^ and MOV10.^36^ All together, our data suggest that many ivRBPs are incorporated into the virion through selective mechanisms.

### The HIV-1 ivRNP heavily overlaps with the Rev protein-protein interactome

**Figure 3:**
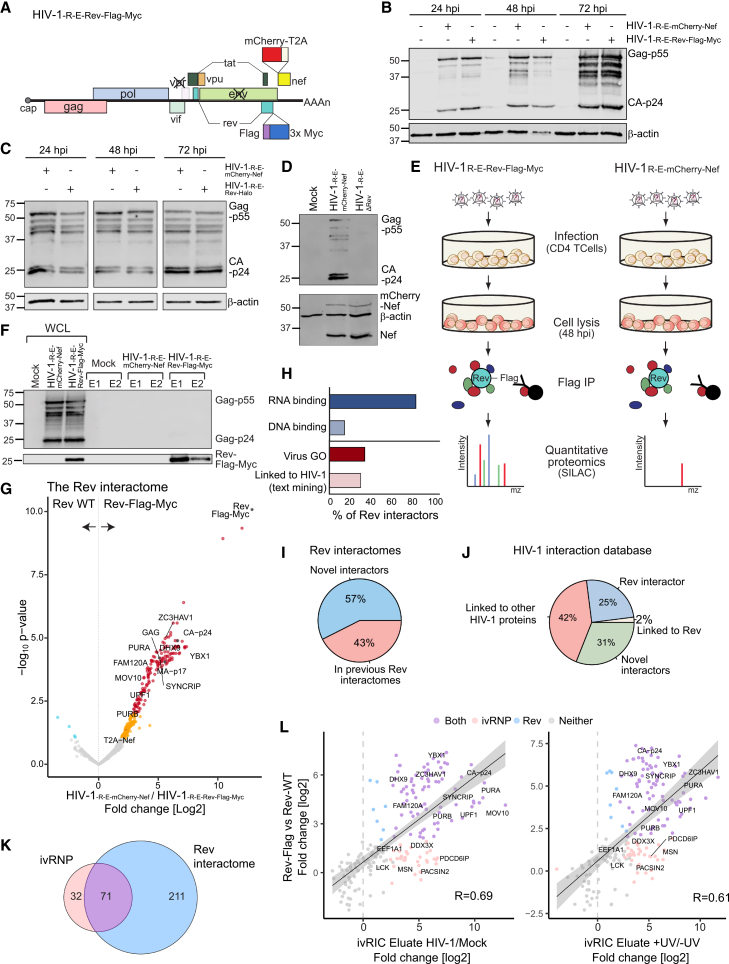
The Rev protein-protein interactome and its similarities with the ivRNP (A) Schematic representation of the HIV-1_R-E-Rev-Flag-Myc_ replicon. (B–D) Western blotting showing viral protein expression in CD4^+^ T lymphocytic cells (SupT1) infected with HIV-1_R-E-Rev-Flag-Myc_ (B), HIV-1_R-E-Rev-Halo_ (C), or HIV-1_R-E-ΔRev_ (D) relative to the parental HIV-1_R-E-mCherry-Nef_. *hpi*, hours post-infection; representative gels of *n* = 3. (E) Schematic representation of the Rev protein-protein interaction experiment, using chimeric pseudotyped viruses to infect CD4^+^ T lymphocytic cells (SupT1). (F) Western blot of inputs and eluates of Rev IP using Flag and p24 antibodies. (G)Volcano plot of the Rev-Flag-Myc immunoprecipitation (IP) versus the untagged control (Rev WT); *n* = 3. Red and dark blue dots are proteins enriched with 1% FDR, while orange and cyan dots are proteins enriched with 10% FDR. Gray dots are non-enriched proteins. (H) Bar plots showing the proportion of Rev interactors annotated with RNA- and DNA-binding (GO terms); virus-related (GO terms), and HIV-1-related (text-mining) functions. (I) Proportion of novel and previously reported Rev interactors in the Rev interactome. (J) Pie chart showing the proportion of proteins classified as novel Rev interactors and previous annotations in HIV-1 NCBI database. (K) Venn diagram showing the overlapping between the ivRIC (ivRNP) and the Rev IP (Rev interactome) experiments. (L) Scatterplots comparing fold changes in the Rev interactome and the ivRIC experiment. Related to Figures S4, S5 and S6.

It was surprising to find that many ivRBPs were nuclear, despite virion assembly occurring at the plasma membrane (Figure 2A). To test if nuclear ivRBPs associate with the gRNA during its nuclear life, we analyzed the interactome of the viral RBP Rev as a proxy for the nuclear gRNP. There have been several attempts at establishing the Rev interactome.^37^^,^^38^^,^^39^ However, resulting datasets had a very poor overlap due to technical limitations (Figure S4A).^2^^,^^37^^,^^38^^,^^39^ To study the Rev interactome in infected CD4^+^ T lymphocytic cells and in the context of HIV-1 infection, we generated a chimeric HIV-1 replicon that expressed Rev fused to Flag-Myc (HIV-1_-R-E-Rev-Flag-Myc_) or HaLo tag (HIV-1_-R-E-Rev-HaLo_) (Figure 3A and S4B). Infection of SupT1 cells with pseudotyped HIV-1 particles led to normal levels of Gag-p55, processed CA-p24, and mCherry-Nef, when compared to the parental HIV-1_-R-E-mCherry-Nef_ (Figures 3B and 3C). By contrast, nullification of Rev by adding a stop codon (HIV-1_-R-E-ΔRev_) led to undetectable levels of Gag-p55 (Figure 3D). Single molecule *in situ* RNA hybridization (smFISH) of cells infected with HIV-1_-R-E-Rev-HaLo_ revealed a normal cytoplasmic accumulation of HIV-1 gRNA when compared to the strictly nuclear distribution observed in HIV-1_-R-E-ΔRev_ (Figure S4C, upper panels). Rev exhibits a nucleolar localization when overexpressed in non-infected cells.^40^ Conversely, expression of Rev from the viral genome led to additional localization in nuclear pores and nucleoplasm (Figure S4C, bottom panels), which is compatible with its known role in nuclear export of HIV-1 RNAs.^2^ These additional localizations support the establishment of native protein-protein interactions involved in viral RNA localization and metabolism.

To elucidate the Rev interactome, we infected SupT1 cells with the pseudotyped HIV-1_-R-E-Rev-Flag-Myc_, followed by Flag immunoprecipitation (IP) at 48 h post-infection (hpi) (Figure 3E). The IP was performed in presence of RNases to minimize the incidence of RNA-bridged interactions. While RNases will reduce the incidence of RNA-dependent interactions, the IP was performed in native conditions, which is expected to co-purify Rev direct binders as well as proteins interacting with it indirectly (e.g., through a complex protein-protein interaction network). Proteomic analysis revealed high sample correlation for Rev-Flag IP eluates, with Rev being the most enriched protein (Figures 3F, 3G, and S4D–S4F; Table S3). We identified 13 peptides mapping unambiguously to Rev across its protein sequence, confirming its expression and sequence identity. Two hundred eighty-four cellular proteins were significantly enriched in Rev IP eluates. Eighty-one percent of the interactors were RBPs themselves (Figure 3H), reflecting Rev’s prominent role in viral RNA metabolism. We noticed the presence of many proteins from the transcriptional apparatus, spliceosome, and ribosome in the IP eluates (Figure S5A and S5B), which is consistent with the observed nucleoplasmatic and nucleolar location of Rev. The previously established Rev interactomes showed very poor overlap^2^; however, 43% of the proteins identified here were also reported in these earlier studies (Figure 3I). Our results thus reconcile the contradictions in these datasets, while still adding many additional Rev interactors. We also observed a strong overlap between our Rev interactome and the annotation in the NCBI HIV database, with 25% being annotated as Rev interactors and a further 44% previously linked to HIV-1 in some capacity (Figure 3J).

To determine to what extent the ivRNP and nuclear gRNP are similar, we compared the ivRIC and Rev IP results. Surprisingly, 68% of the components of the ivRNP are also interactors of Rev, displaying similar fold changes in both datasets that suggest analogous stoichiometry (R > 0.61; Figures 3K–3L). Proteins shared between these two datasets are mostly involved in RNA transcription, splicing, nuclear export, and translation (Figure S5C). By contrast, proteins only present in the ivRNP are associated mainly with membrane biology, including membrane trafficking, endocytosis, and vesicle transport. Our results are thus compatible with two populations of ivRBPs: one that associates with the gRNA in the nucleus and remains associated with it, and another that engages with the gRNA later at the plasma membrane. Interestingly, we also observed that the previously established Gag^41^ and Staufen^42^ interactomes (proxies for the cytoplasmic gRNPs) overlap to some extent with the ivRNP and the Rev interactome (Figure S6A). Moreover, we found a moderate overlap with intracellular HIV-1 RNA interactomes (Figure S6B).^10^^,^^43^

### PURA and PURB are regulators of HIV-1 particle infectivity

To assess if ivRBPs are functionally relevant, we generated knockouts (KOs) in CD4^+^ T lymphocytic cells (SupT1) for the transcriptional activator protein Pur-alpha (PURA), its homolog Pur-beta (PURB) and FAM120A, which were identified by both ivRIC and Rev protein-protein interaction analyses. We also selected moesin (MSN) that is a membrane-associated protein and was only present in the ivRNP. No major effects in cell viability, proliferation, or cytotoxicity were detected in KO cells (Figures S7A–S7D).

**Figure 4:**
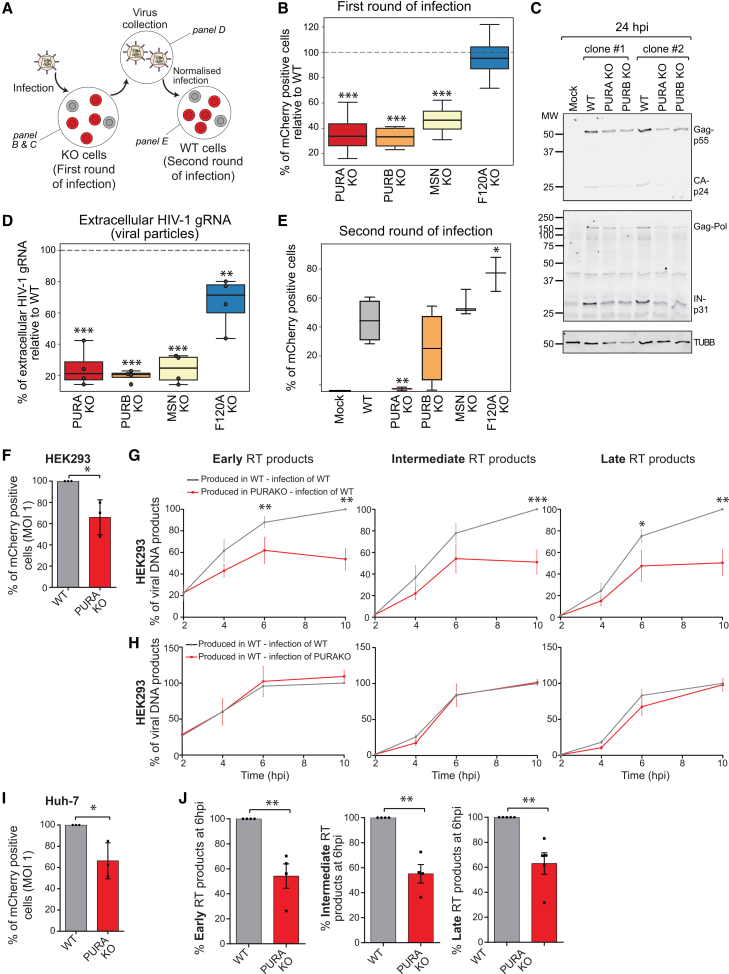
Effect of ivRBPs in viral gene expression and virion production and infectivity (A) Representation of the experimental design. (B) Flow cytometry analysis of WT and KO SupT1 cells infected with HIV-1_mCherry-Nef_ for 48 h. *y* axis show the percentage of KO cells expressing mCherry as compared to WT cells (dashed line). (C) Western blotting analysis of WT, PURA, and PURB KO SupT1 cells infected with HIV-1_mCherry-Nef_ for 24 h; a representative gel of *n* = 3. (D) RT-qPCR analysis of the HIV-1 gRNA in the supernatant of WT- and KO-infected SupT1 cells from (B). (E) Viral particles purified in (D) from KO and WT cells were used to infect WT SupT1 cells upon normalization by gRNA levels. mCherry positive cells were quantified by flow cytometry at 48 hpi. For (B), (D), and (E), *n* = 4; ^∗^*p* < 0.05; ^∗∗^*p* < 0.01; ^∗∗∗^*p* < 0.001. (B), (D), and (E) show box-and-whisker plots, in which the boxes represent the interquartile range (Q1–Q3) with the median indicated, and the whiskers denote the minimum and maximum values. (F) Flow cytometry analysis of HEK293 WT cells infected at MOI 1 with single round HIV-1_R-E-mCherry-Nef_ produced from HEK293 Flp-In T-Rex WT or PURA KO cells; *n* = 3. (G) Analysis of early, intermediate, and late reverse transcription (RT) products in HEK293 Flp-In T-Rex WT cells infected with normalized single round HIV-1_R-EmCherry-Nef_ particles produced in HEK293 Flp-In T-Rex WT or PURA KO cells; *n* ≥ 3. (H) Analysis of early, intermediate, and late HIV-1 RT products in HEK293 Flp-In T-Rex WT or PURA KO cells infected with normalized single round HIV-1_R-E-mCherry-Nef_ particles produced in WT cells; *n* = 4. (I) Flow cytometry analysis of HEK293 Flp-In T-Rex WT cells infected with normalized single round HIV-1_R-E-mCherry-Nef_ particles produced in Huh-7 WT or PURA KO cells; *n* = 3. (J) Analysis of early, intermediate, and late RT products at 8 hpi in HEK293 Flp-In T-Rex WT cells infected with HIV-1_R-E-mCherry-Nef_ produced in Huh-7 WT or PURA KO cells; *n* ≥ 4. (F–J) Data is mean ± standard error of the mean (SEM), including individual data points. For all panels, ∗*p* < 0.05, ∗∗*p* < 0.01. Related to Figures S7 and S8.

Infection of CD4^+^ T lymphocytic KO cells with HIV-1_mCherry-Nef_ infection led in all cases, except for FAM120A, to a reduction in the number of mCherry-positive cells (Figures 4A, 4B, and S7E), suggesting that PURA, PURB, and MSN are important for viral gene expression and/or spread in CD4^+^ T lymphocytic cells. The effects of PURA and PURB on HIV-1 gene expression were also observed by western blot (Figures 4C, S7F, and S7G). Previous work linked PURA to HIV-1 transcription, but this work was mostly done by protein overexpression and using reporter genes.^44^^,^^45^ We applied smFISH to PURA KO HEK293 cells infected with HIV-1_R-E-Gag-mCherry_ and observed an overall reduction in HIV-1 RNA molecules, in both the nucleus and cytoplasm, that correlates with a reduction in Gag-derived mCherry signal (Figures S7H and S7I). Strikingly, HIV-1 nuclear transcription foci had similar fluorescence intensity in WT and KO cells, but their number exhibited a non-significant but substantial decrease in PURA KO cells (Figure S7I). This suggests that PURA’s roles in HIV-1 infected cells involves transcription activation and not elongation, in agreement with previous results.^44^ The concurrence of our results with the known function of PURA in HIV-1 cells reinforces the validity of our cell lines.

To determine if viral particle production is affected by the absence of PURA, PURB, MSN, or FAM120A, we collected the supernatant of the infected SupT1 KO cells and quantified it by RT-qPCR (Figures 4A and 4D). We observed a remarkable decrease of HIV-1 gRNA in the supernatant of KO cells, which correlated well with the reduced infection observed by flow cytometry (Figures 4B vs. 4D) as well as the transcription activation defects observed by smFISH (Figure S7I). These results confirmed the importance of PURA, PURB, and MSN in the HIV-1 life cycle by an orthogonal approach.

We next tested if ivRBPs are important for HIV-1 infectivity by infecting WT cells with the same number of viral particles produced in WT or KO cells (Figure 4A). Viruses lacking MSN or FAM120A exhibited nearly no differences in infectivity compared to a virus produced in WT cells (Figure 4E). By contrast, viruses lacking PURA and PURB produced a substantially lower number of HIV-1-positive WT cells (Figure 4E). All together, our results suggest that ivRBPs can act at several stages of the HIV-1 life cycle, with PURA and PURB having roles in HIV-1 viral transcription and viral particle infectivity.

To validate these results, we generated HIV-1_R-E-mcherry-Nef_ viruses in HEK293 WT or PURA KO cells and, after normalization by CA-p24 abundance, we infected HEK293 WT cells. In agreement with the experiments in T CD4^+^ lymphocytic cells, the number of mCherry-positive cells was significantly lower with viruses produced in PURA KO cells than in WT cells (Figure 4F). We collected samples at various times post-infection and analyzed by PCR the presence of early, intermediate, and late HIV-1 reverse transcription products. Strikingly, we observed a significant reduction of the three types of reverse transcription products when infecting with viruses produced in PURA KO cells compared to those produced in WT cells (Figure 4G). To evaluate the requirement of PURA inside HIV-1 particles, we infected PURA KO cells with viruses produced in WT cells (i.e., containing PURA inside virions). Notably, we observed no differences in reverse transcription, highlighting that the defects depend upon the presence of PURA inside the virions (Figure 4H). Infectivity and reverse transcription defects also occurred with HIV-1_R-E-mcherry-Nef_ generated in Huh-7 PURA KO cells (Figures 4I and 4J), demonstrating reproducibility across three cell lines (SupT1, HEK293 and Huh7).

### PURA and PURB interact with HIV-1 proteins and gRNA

**Figure 5:**
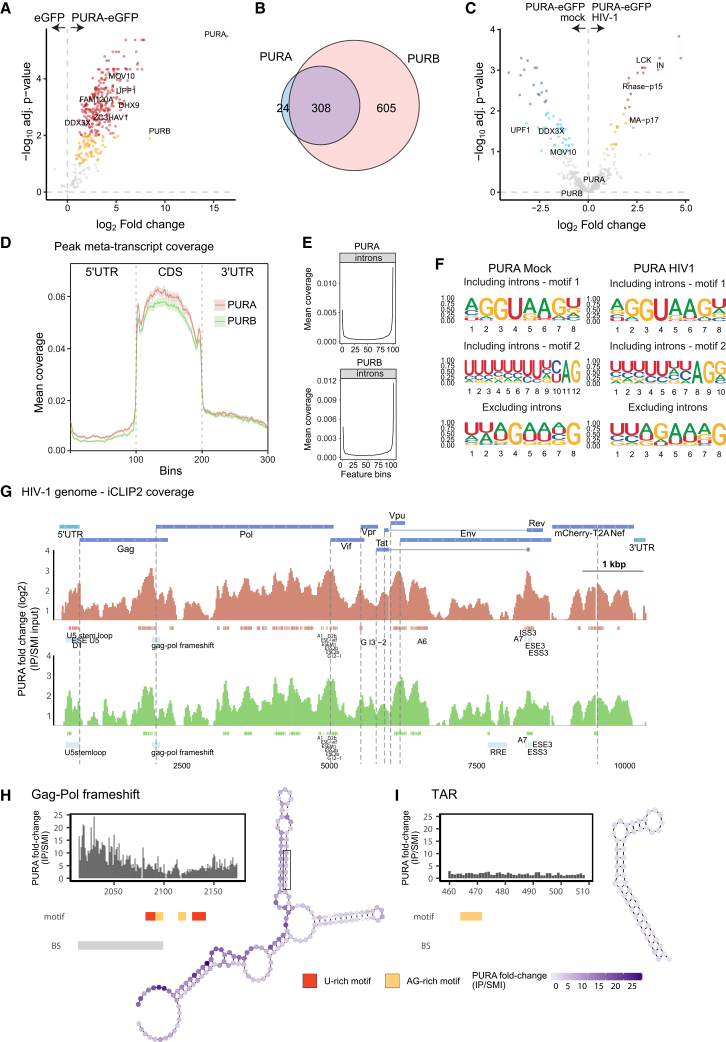
Analysis of the interactions that PURA and PURB establish in CD4^+^ lymphocytic cells (A) Volcano plot showing the proteins enriched in PURA-EGFP IP over the EGFP control IP in Jurkat Flp-In T-REx cells expressing these proteins. Dots represent proteins enriched with 1% FDR (red and dark blue) or with 10% FDR (orange and cyan). Gray dots are non-enriched proteins; *n* = 3. (B) Venn diagram comparing the proteins that are enriched in both PURA-EGFP and PURB-EGFP IPs. (C) Volcano plot showing the proteins enriched in PURA-EGFP IP in mock versus HIV-1 infected cells. Color code as in (A). (D) iCLIP2 (*n* = 3) was applied to Jurkat Flp-In T-REx expressing PURA-EGFP or PURB-EGFP and infected with HIV-1_R-E-mCherry-Nef_ or mock-infected. Density plot showing the distribution of binding sites across 5′ UTRs, CDSs and 3′ UTRs of target RNAs. (E) As in (D) but for introns. (F) Motifs enriched in RNA binding sites of PURA including or excluding intronic sequences. (G) Binding profile of PURA-EGFP and PURB-EGFP on HIV-1 RNA. Significant binding sites (*p* < 0.01) are indicated with salmon (for PURA) or green (for PURB) boxes underneath the density plot. (H and I) Binding site density at the Gag-Pol frameshift (H) and TAR (I). The significant (*p* < 0.01) binding sites are indicated with a gray box, while the AG- and U-rich motifs are indicated with yellow and red boxes, respectively. The secondary structure of these RNA elements was generated using SHAPE data,^46^ indicating the fold change in the IP over the SMI control at each nucleotide position. Related to Figures S9 and S10.

Our next step was to determine the scope of cellular and viral proteins that associate with PURA/B. To do so, we generated lymphocytic Jurkat Flp-In T-REx cells able to express PURA or PURB fused to EGFP in a doxycycline-dependent manner (Tet-on) (Figures S7J and S7K). IP of PURA-EGFP and PURB-EGFP with GFP-Trap revealed a specific enrichment of both baits (Figures 5A and S8A–S8C). Proteomic analysis revealed hundreds of proteins, including many ivRBPs, co-precipitating with PURA-EGFP and PURB-EGFP (Figures 5A and S8C; Table S4). The interactome of the two proteins largely overlapped and exhibited high protein intensity correlation, implying similar protein composition and stoichiometries (Figures 5B and S8D). PURB was the protein with highest fold change in PURA-EGFP IP and vice versa (Figures 5A and S8C), indicating that both proteins associate in heteromeric complexes. Both PUR proteins interact with numerous components of the splicing, nuclear RNA export, and translation machinery (Figure S8E), suggesting their participation at multiple steps of the RNA life cycle. Many proteins exhibited differential association with PURA and PURB upon HIV-1 infection (Figures 5C, S8C, and S8F), including viral proteins MA, IN, and the RNaseH-p15 that were present in both interactomes. These interactions support a potential involvement of PURA/B in processes that dictate the ability of HIV-1 virions at establishing infection, including reverse transcription (Figure 4G) and/or integration.

To validate the interaction of PURA with HIV-1 gRNA inside HIV-1 virions, we transfected HIV-1_R-E-mCherry-Nef_ in 293 Flp-In T-REx cells expressing unfused EGFP or PURA-EGFP. Resulting particles were purified and subjected to IP with an anti-EGFP nanobody. Presence of HIV-1 gRNA in eluates was determined by RT-qPCR. Importantly, we observed ∼10-fold enrichment in viral gRNA in PURA-EGFP IP eluates over EGFP (Figure S8G), further confirming PURA as ivRBP by an orthogonal method.

To define the position(s) within the gRNA where binding occurs, we applied iCLIP2^47^ to infected and uninfected PURA-EGFP and PURB-EGFP Jurkat cells. Both proteins were strongly enriched in IP eluates, forming detectable protein-RNA complexes with decreased mobility in SDS-PAGE (Figures S9A–S9C). We used size-matched inputs (SMIs) and unfused EGFP as control for the iCLIP2 analysis, and we performed standard quality assessments (Figures S9D–S9E). PURA/B interact predominantly with mRNAs, particularly near the 5′ end and the start and stop codons (Figures 5D and S9F–S9H; Table S5). We also observed sharp binding peaks at the 5′ and 3′ splice sites of introns (Figure 5E). The RNA targets and binding sites of PURA and PURB largely overlap, resulting in clustering in PCA analysis (Figures 5D, 5E, S9F–S9I, and S10A). The extensive overlapping of PURA and PURB binding sites further supports the existence of heteromeric complexes.

We next tested if PURA/B interact with specific sequences using motif enrichment tools, revealing two prominent binding motifs: a purine-rich sequence that is consistent with the well-known binding motif of PURA,^48^ preceded by a previously undetected U-rich motif (Figures 5F, S10B, and S10C). These motifs strongly resemble the sequences found at splice sites.^49^ To rule out that these motifs are artifact derived from the proximity of splicing junctions to PURA/B binding sites, we searched again for motifs after removal of all intronic sequences. A single motif combining the U-rich sequence followed by the AG-rich sequence was observed (Figures 5F and S10B), validating PURA/B binding to the observed bi-partite motif.

We next tested if PURA/B interact with viral RNA. Our results revealed that a substantial proportion of the reads (5%–20%) map to the HIV-1 gRNA in infected cells (Figures S10D and S10E). Binding sites distributed across the gRNA overlapping with important regulatory elements, including most canonical splice sites, which contained in most cases the AG-rich or/and U-rich motifs (Figures 5G and S10F–S10K). Similar read density distribution across the splice sites suggests that PURA/B binds primarily to the gRNA. Moreover, a prominent PURA/B binding site preceded the slippery sequence that causes the Gag/Pol frameshift, which contained two repeats of the AG- and U-rich motifs (Figure 5H). However, the Gag/Pol ratio was not affected in PURA and PURB KO cells, suggesting that these proteins do not affect frameshift efficiency (Figure S7G). Early biochemical assays proposed that PURA could interact with the TAR structure in the HIV-1 genome to regulate Tat-dependent transcription.^44^^,^^45^ iCLIP2 did not reveal binding sites proximal to the TAR structure *in cellulo* (Figure 5I). However, we detected binding to other regions of the 5′ UTR containing important regulatory structures (Figure 5G). Both PURA and PURB displayed nearly identical binding distribution across the gRNA, except for a binding site of PURB nearby the Rev response element (RRE) that was not detected for PURA (Figures 5G and S10E). An additional binding site immediately downstream the RRE that overlaps with several 3′ splice sites was detected for both proteins. Binding to RRE and its proximities is compatible with the co-purification of PURA/B with Rev. All together, these results reveal a widespread binding of PURA/B across the gRNA that is compatible with their high levels in the ivRNP (Figures 1B and 2G).

## Discussion

Many approaches have been applied to profile the composition of viral particles.^9^ However, the fact that virions assemble in the crowded cellular environment is compatible with passive incorporation of cellular proteins with no roles in the viral life cycle. Viral particle proteomes are usually large and exhibit little consistency between studies, which complicates the distinction between incidental bystander proteins and those that are functionally relevant for the viral particles. Our study shows that the total HIV-1 particle proteome (ivRIC input) correlates well with protein abundance in the producer cell. By contrast, ivRIC underscores the proteins that engage with the HIV-1 genome within virions. The reduced correlation of the identified proteins with the proteome of the producer cell supports a model in which the incorporation of genome-bound proteins occur through active mechanisms. Incorporation of these RBPs into virions likely involves specific binding to HIV-1 gRNA or/and proteins. Importantly, several ivRBPs have been implicated in HIV-1 particle formation or infectivity by independent studies,^26^^,^^27^^,^^28^^,^^29^^,^^30^^,^^31^^,^^32^^,^^33^^,^^34^^,^^50^ supporting the high quality of our results. Strikingly, we observed a pronounced overlap between the ivRNP and the Rev interactome, suggesting a fascinating connection between the nuclear life of the HIV-1 gRNA and the formation of the viral particles. HIV-1 gRNA is produced by the cellular RNA polymerase II, and it is processed in the cell’s nucleus as other cellular mRNAs, including the addition of the cap and poly(A) tail. However, the gRNA is “perceived” by the host cell as immature, as it contains intronic sequences, leading to nuclear retention.^2^ The viral protein Rev is produced by a multiple spliced RNA that is exported to the cytoplasm by a conventional mRNA pathway. Rev accumulates over time and travels to the nucleus, where it is crucial for the engagement with the CRM1 RNA export pathway for nuclear HIV-1 gRNA export.^2^ Once in the cytoplasm, the gRNA can be translated or packaged into viral particles. Evidence suggests the existence of two structurally distinct populations of gRNAs, generated by differential transcription initiation, that are distinguished by the number of 5′ terminal guanosines.^51^^,^^52^^,^^53^^,^^54^^,^^55^ gRNAs with a single guanosine are prone to cap sequestration, forming a secondary structure conducive to gRNA dimerization and packaging. Conversely, gRNAs with two or three terminal guanosines exhibit higher translational efficiency due to greater accessibility of the cap structure.^51^^,^^52^^,^^53^^,^^54^^,^^55^ Consequently, virion-packaged gRNAs predominantly contain a single 5′ terminal guanosine and are less likely to be translated. Given that translation factors and ribosomes play a crucial role in remodeling RNPs during translation initiation and elongation,^56^^,^^57^ it is anticipated that lack of translation of virion-packaged gRNAs will lead to retention of nuclear RBPs. This phenomenon could explain the similarity between virion-packaged RNPs and the Rev interactome, reflecting the nuclear history of the gRNA.

In the early 2000s, PURA was linked to Tat-dependent transcription using LTR-controlled plasmids.^44^ However, these early publications were contested by subsequent studies, as reviewed in detail elsewhere.^58^ It was suggested that the use of PURA constructs that violated domain boundaries (defined by later resolved crystal structures) could lead to misfolded dysfunctional proteins. Additionally, the experiment conducted made it difficult to separate between transcriptional and post-transcriptional effects of PURA.^44^^,^^45^ Our single-molecule RNA analyses now show that provirus activation is partially inhibited in cells lacking PURA and that a lower number of transcriptionally active proviruses cause a reduction in gRNA. Additionally, we also show that both PURA and PURB interact with transcriptional and post-transcriptional machineries, as well as with key post-transcriptional regulatory elements on the HIV-1 gRNA, suggesting additional roles for PURA beyond HIV-1 transcription, for example in HIV-1 splicing given its binding to splice sites. We also show a new function of PURA and PURB in the viral particles, affecting their infectivity. The ivRBPs DHX9 and EEF1A were proposed to modulate reverse transcription through its interaction with the RT.^32^^,^^33^ PURA and PURB co-precipitate with several components of the Gag-Pol polyprotein, including MA, RT, the RNase H domain (p15) and IN. Our results show that loss of PURA causes an inhibition of reverse transcription perhaps through its interaction with RNaseH-p15 or indirectly through its binding to the gRNA. Our results suggest that the regulatory roles of cellular proteins such as PURA and PURB may span different stages of HIV-1 infection, affecting HIV-1 gene expression and viral particle infectivity.

ivRIC was applied here to HIV-1 viral particles generated in the T CD4^+^ lymphocytic SupT1 cell line. However, variations in the cellular proteome configuration can lead to different ivRBPs being incorporated into virions. This intriguing possibility warrants further investigation using various producer cells, such as primary T CD4^+^ lymphocytes and macrophages. Additionally, the impact of cell-dependent changes in the composition of virion-packaged gRNPs on the properties of viral particles should be thoroughly examined. These analyses will offer fresh perspectives on the cellular proteins that govern the functionality of viral particles.

### Limitations of the study

ivRIC employs UV light to establish “zero distance” RNA-to-protein cross-linking. While highly specific, UV cross-linking suffers from low efficiency, particularly with proteins binding to double-stranded RNA tracts. To achieve a full picture of the virion-packaged gRNP, other cross-linkers such as formaldehyde could be used, acknowledging that chemical cross-linkers such as formaldehyde have lower specificity as they can also mediate longer distance covalent bonds between proteins.^59^^,^^60^ Here, we used oligo(dT) for viral RNA enrichment because HIV-1 gRNA has a poly(A) tail. To target other viruses with no poly(A) tail, it is possible to use specific antisense probes or total RNA purification methods.^59^^,^^60^ Additional quality controls, such as treatment of purified particles with RNases, will be required to rule out interactions with extra-virion RNA, particularly in experiments where the proportion of isolated viral RNA over cellular RNA is lower than with HIV-1. Such experiments would provide unambiguous evidence that the binding of ivRBPs to the isolated RNA occurs within the viral capsid. As with any proteomics-based method, protein identification can be affected by protein abundance, length, and amino acid composition, potentially resulting in false negatives. We optimized the amount of input viral particles used in ivRIC to achieve a comprehensive analysis of the gRNP packaged in HIV-1 virions. However, other viruses may require larger starting material to achieve similar depth, which will depend upon the number of viral RNA molecules per particle, the efficiency of the viral RNA purification and the sensitivity of the downstream mass spectrometry. Therefore, optimization and pilot experiments are likely required if applying ivRIC to other viruses. Several key control samples and quality control checks were used here to account for all potential sources of contamination, as described in Figures 1 and S1. If applying ivRIC to another viral model, these control samples and analysis (and others if relevant) should be put in place to confirm data quality.

## Resource availability

### Lead contact

Further information and requests for resources and reagents should be directed to and will be fulfilled by the lead contact, Alfredo Castello (alfredo.castello@glasgow.ac.uk).

### Materials availability

All unique reagents and materials generated for this study, including plasmids and engineered cell lines, are available from the lead contact upon reasonable request and in accordance with the standard material transfer procedures of the University of Glasgow. This study generated plasmids and engineered cell lines that are derived from commercially available vectors and cell lines subjected to third-party licensing restrictions. For this reason, these materials have not been deposited in public repositories. Requests for materials should be directed to the lead contact.

### Data and code availability

• All proteomics data generated in this study have been deposited in the PRIDE database under accession number PRIDE: PXD042829. iCLIP2 RNA sequencing data have been deposited in the Gene Expression Omnibus (GEO) under accession number GEO: GSE262435. RNA sequencing data from purified HIV-1 particles have been deposited in the European Nucleotide Archive (ENA) under accession number ENA: PRJEB105389.
• This study did not generate original code.
• Any additional information required to reanalyze the data reported in this paper is available from the lead contact upon reasonable request.

## Acknowledgments

A.C. is funded by the European Research Council (ERC) Consolidator Grant “vRNP-capture” no. 101001634, the Career Development Award #MR/L019434/1, the John Fell Funds from the University of Oxford, and the Medical Research Council (MRC) grants MR/R021562/1 and MC_UU_00034/2. M.G.-M. is funded by the European Union’s Horizon 2020 research and innovation program under the Marie-Sklodowska-Curie grant agreement no. 700184. R.T. is funded by a BBSRC DTP scholarship DD01.20. L.I. is funded by the Biotechnology and Biological Sciences Research Council (BBSRC) DTP scholarship no. BB/M011224/1. C.E.L. is funded by the Department of Biochemistry Graduate Scholarship and the Hertford College Graduate Studies Scholarship of the University of Oxford. A.E.-B. is funded by Fundación Ramón Areces Post-doctoral fellowship program. T.J.M.S. is funded by Fondation pour la Recherche Médicale (FRM - FDT202001010798). W.K. is funded by the European Union’s Horizon 2020 research and innovation program under Marie-Sklodowska-Curie no. 842067. Q.G. was funded by the UK MRC MC_UU_00034/5. V.R. is funded by the European Union’s Horizon 2020 research and innovation program under Marie-Sklodowska-Curie no. 892756. E.P.R. is funded by Agence Nationale des Recherches sur le SIDA et les Hépatites Virales (ANRS – ECTZ3306), Fondation Finovi, and the ERC (ERC-StG-LS6-805500) under the European Union’s Horizon 2020 research and innovation program as well as the ATIP-Avenir program. I.D. was funded by the Wellcome Trust Investigator Award 209412/Z/17/Z and the University of Glasgow.

While this work is funded by the European Union, views and opinions expressed are however those of the author(s) only and do not necessarily reflect those of the European Union or the European Research Council Executive Agency. Neither the European Union nor the granting authority can be held responsible for them.

## Author contributions

Conception/design, M.G.-M., A.E.-B., R.T., and A.C.; data acquisition, M.G.-M., A.E.-B., R.T., C.E.L., H.C., M.N., T.J.M.S., K.D., S.D.S., W.K., N.P., V.R., and S.H.; data analysis, M.G.-M., R.T., L.I., C.E.L., H.C., S.D.S., Q.G., A.I.J., W.K., J.Y.L., I.D., S.M., and A.C.; data interpretation, M.G.-M., A.E.-B., R.T., L.I., J.Y.L., I.D., M.N., S.M., and A.C.; writing – original draft, A.C.; writing – editing, all authors; funding acquisition, M.G.-M., A.E.-B., I.D., S.M., and A.C.; resources, T.J.M.S., J.Y.L., M.N., E.P.R., I.D., S.M., and A.C.; supervision, M.G.-M., M.N., E.P.R., I.D., S.M., and A.C.

## Declaration of interests

The authors declare no competing interests.

## STAR★Methods

### Key resources table

**Table:** 

REAGENT or RESOURCE	SOURCE	IDENTIFIER
**OMICS Data**		

Dataset	Repository	ID
Proteomics data	PRIDE	PXD042829
iCLIP2 data	GEO	GSE262435
RNAseq of purified HIV particles	ENA	PRJEB105389

**Antibodies**		

Human monoclonal HIV-1 p24	NIBSC – Center for AIDS Reagents	Cat#ARP3279
Mouse monoclonal HIV-1 Integrase (IN-2)	Santa Cruz	Cat#sc-69721
Rabbit HIV-1 gp120	NIBSC – Center for AIDS Reagents	Cat#ARP421
Mouse monoclonal HIV-1 Nef (clone 3D12)	NIBSC – Center for AIDS Reagents	Cat#EVA3067.5
Rabbit polyclonal MOV10	Cusabio	Cat#CSB-PA862068LA01HU
Rabbit polyclonal PURA	Abcam	Cat#ab79936
Rabbit polyclonal PURA	Proteintech	Cat#17733-1-AP
Rabbit polyclonal SUB1	Abcepta	Cat#AP19807c-ev
Mouse monoclonal β-ACTIN (clone AC-15)	Merck	Cat#A1978
Rabbit monoclonal Myc-Tag (71D10)	Cell Signaling	Cat#2278S
Rabbit polyclonal PURB	Proteintech	Cat#18128-1-AP
Rabbit polyclonal FAM120A	ProSci	Cat#5307
Rabbit polyclonal MSN (clone FIC-13)	Abcepta	Cat#AP13752b
Rat monoclonal GFP (3H9)	ChromoTek GmbH	Cat#3h9-100
Mouse monoclonal Tubulin (clone DM1A)	Sigma Aldrich	Cat#T9026
Rabbit monoclonal PTPRC/CD45 (clone 6O19)	Thermo Fisher Scientific	Cat#80297-1-RR100UL
Mouse monoclonal SPN/CD43 (clone 2A11D6)	Proteintech	Cat#66224-1-Ig
Rabbit polyclonal SERINC3	Proteintech	Cat#20267-1-AP
Mouse monoclonal TetR (clone 9G9)	Takara Bio	Cat#631131
IRDye 680RD Goat anti-Human IgG Secondary Antibody	Li-Cor	Cat#926-68078
IRDye 800CW Goat anti-Human IgG Secondary Antibody	Li-Cor	Cat#926-32232
IRDye 680RD Donkey anti-Mouse IgG Secondary Antibody	Li-Cor	Cat#926-68072
IRDye 800CW Donkey anti-Mouse IgG Secondary Antibody	Li-Cor	Cat#926-32212
IRDye 680RD Donkey anti-Rabbit IgG Secondary Antibody	Li-Cor	Cat#926-68073
IRDye 800CW Donkey anti-Rabbit IgG Secondary Antibody	Li-Cor	Cat#926-32213
IRDye 680RD Goat anti-Rat IgG Secondary Antibody	Li-Cor	Cat#926-68076
IRDye 800CW Goat anti-Rat IgG Secondary Antibody	Li-Cor	Cat#926-32219

**Chemicals, peptides, and recombinant proteins**		

Janelia Fluore Halo-646 ligand	Promega	Cat#GA1120
Cell Mask	Invitrogen	Cat#C37608
3X FLAG peptide	Merck	Cat#F4799-4MG

**Critical commercial assays**		

Oligo(dT)25 beads	New England Biolabs	Cat#S1419S
Luna Universal One-Step RT-qPCR kit	New England Biolabs	Cat#E3005S
HiScribe T7 ARCA mRNA kit with tailing	New England Biolabs	Cat#E2060S
HIV-1 Gag p24 DuoSet ELISA kit	Bio-Techne	Cat#DY7360-05
NextSeq500/NextSeq 550	Illumina	Cat#20024907/20024906
anti-FLAG M2 magnetic beads	Merck	Cat#M8823-1ML
GFP_Trap agarose bead slurry	ChromoTek	Cat#gta

**Deposited data**		

Proteomic datasets	PRIDE	PXD042829
RNA sequencing data (iCLIP2)	GEO	GSE262435
Viral particle RNAseq	ENA	PRJEB105389
Human proteome reference (downloaded Nov 2016)	UniProt	UP000005640
Human genome (Release 104)	ENSEMBL	GRCh38
RBPbase v0.23 alpha	EMBL	https://rbpbase.shiny.embl.de/

**Experimental models: Cell lines**		

SupT1	ECACC	Cat#95013123; RRID:CVCL_1714
SupT1 PURA KO; PURB KO; MSN KO; FAM120A KO	This paper	N/A
Flp-In Jurkat	Thermo Fisher Scientific	Cat#R76207; RRID:CVCL_U426
Flp-In T-Rex Jurkat	This paper	N/A
Flp-In T-Rex Jurkat EGFP; PURA-EGFP; PURB-EGFP	This paper	N/A
Flp-In T-Rex 293	Thermo Fisher Scientific	Cat#R78007
Flp-In T-Rex 293 PURA-EGFP	This paper	N/A
Flp-In T-Rex 293 PURA KO	This paper	N/A
HeLa	ATCC	Cat#CCL-2
HEK293T	ATCC	Cat#CRL-3216
Huh-7	–	Prof. Matthias Hentze (gift)
Huh-7 PURA KO	Yeh et al.^61^	Dr. Mariano A Garcia-Blanco (gift)

**Oligonucleotides**		

(RT-qPCR) HIV genomic qPCR F: ctgaagcgcgcacggcaa	–	N/A
(RT-qPCR) HIV genomic qPCR R: gacgctctcgcacccatctc	–	N/A
(Early RT) hRU5-F: GCCTCAATAAAGCTTGCCTTGA	–	N/A
(Early RT) hRU5-R: TGACTAAAAGGGTCTGAGGGATCT	–	N/A
(Intermediate RT) Gag-F1: CTAGAACGATTCGCAGTTAATCCT	–	N/A
(Intermediate RT) Gag-R1: CTATCCTTTGATGCACACAATAGAG	–	N/A
(Late RT) MH531: TGTGTGCCCGTCTGT TGTGT	–	N/A
(Late RT) MH532: GAGTCCTGCGTCGAG AGATC	–	N/A
GAPDH –F: ATGGGGAAGGTGAAGGTCG	–	N/A
GAPDH –R: GGGGTCATTGATGGC AACAATA	–	N/A
Primers for cloning, sgRNAs and knock out validation	This paper	See Table S6

**Recombinant DNA**		

pNL4-3	NIBSC – Center for AIDS Reagents	Cat#2006
pNL4-3.Luc.R-E−	NIBSC – Center for AIDS Reagents	Cat#2128
pNL4.3-mCherry-T2A-Nef	This paper	N/A
pNL4-3.R-E-mCherry-T2A-Nef	Garcia-Moreno et al.^62^	N/A
pNL4-3.R-E-mCherry-T2A-Nef-Rev-Flag-3xMyc	This paper	N/A
pNL4-3.R-E-mCherry-T2A-Nef -Rev-Halo	This paper	N/A
pNL4-3.R-E-mCherry-T2A-Nef-ΔRev	This paper	N/A
pNL4.3-mCherry-T2A-gag	Garcia-Moreno et al.^62^	N/A
psPAX-2	Addgene	Cat#12260
pHEF-VSVG	NIH AIDS Reagent Program	Cat#4693
pcR-Blunt vector	Thermo Fisher Scientific	Cat#K270020
pcR-Blunt-NL4.3-mCherry-T2A-Nef	This paper	N/A
pcDNA6/TR plasmid	Thermo Fisher Scientific	Cat#V102520
pOG44	Thermo Fisher Scientific	Cat#V600520
pcDNA5/FRT/TO-EGFP	Castello et al.^63^	N/A
pcDNA5/FRT/TO-EGFP-linker	Garcia-Moreno et al.^62^	N/A
MGC Human PURA Sequence-Verified cDNA (CloneId:5284976)	Horizon	Cat#MHS6278-202808724
BLADE plasmid	Mageot et al.^64^	N/A

**Software and algorithms**		

CFX Manager Software v3.1	Bio-Rad	https://www.bio-rad.com/en-uk/sku/1845000-cfx-manager-software?ID=1845000
ImageJ	NIH	https://imagej.net/ij/
MaxQuant v1.6.3.4	Max-Planck-Institute of Biochemistry	https://www.maxquant.org/
Image Studio Software	LICORbio	https://www.licorbio.com/image-studio
FlowJo	BD Life Sciences	https://www.flowjo.com/flowjo/download
R	The R Foundation	https://www.r-project.org/
BiomaRt	Durinck et al.^65^^,^^66^	https://bioconductor.org/packages/release/bioc/html/biomaRt.html
UpsetR	Conway et al.^67^	https://cran.r-project.org/web/packages/UpSetR/index.html
venneuler	–	https://cran.r-project.org/web/packages/venneuler/index.html
imputeLCMD	–	https://CRAN.R-project.org/package=imputeLCMD
FISH-quant v2	Imbert et al.^68^	–
*limma*	Ritchie et al.^69^	–
STAR aligner	Dobin et al.^70^	–
Cutadapt	–	Cutadapt removes adapter sequences from high-throughput sequencing reads | Martin | EMBnet.journal
Je Suite	Girardot et al.^71^	–
HTSeq-clip	Sahadevan et al.^72^	–
BEDTools	Quinlan and Hall^73^	–
DEW-Seq	Sahadevan et al.^72^	–
GenomicRanges R package	Lawrence et al.^74^	–
BBMap	–	https://sourceforge.net/projects/bbmap/
AnnotationDbi	–	https://bioconductor.org/packages/release/bioc/html/AnnotationDbi.html
HOMER	Heinz et al.^75^	http://homer.ucsd.edu/homer/
universalmotif	Tremblay et al.^76^	https://www.bioconductor.org/packages/release/bioc/html/universalmotif.html
ggseqlogo	Wickam et al.^77^	https://cran.r-project.org/web/packages/ggseqlogo/index.html
ggplot2	Wickam et al.^77^	https://ggplot2.tidyverse.org/
Cytoscape v3.9.1	Shannon et al.^78^	https://cytoscape.org/
stringApp	Docheva et al.^79^	–
clusterMaker2	Morris et al.^78^	–
MCODE	Bader and Hogue^81^	–
DyNet	Goenawan et al.^82^	–
EnrichR	Xie et al.^83^	https://maayanlab.cloud/Enrichr/
REVIGO	Supek et al.^84^	–
TIDE	Brinkman et al.^85^	–
Prism v10	GraphPad	https://www.graphpad.com/features

### Experimental model and study participant details

#### Viruses and cells

The following human cell lines are available in cell culture collections or commercially: T-lymphoblast SupT1 (male; ECAAC, #95013123), T-lymphocyte Jurkat Flp-In (male; Thermo Fisher Scientific, #R76207), embryonic kidney HEK293 Flp-In T-REx (female; Thermo Fisher Scientific, #R78007), HeLa (female; ATCC, #CCL-2), Huh-7 (male; a gift from Matthias W Hentze) and HEK293T (female; ATCC, #CRL-3216). Jurkat Flp-In and tetracycline inducible Jurkat Flp-In T-REx expressing tagged RBPs were obtained as described below. Most cell lines utilised in this study were sourced from reputable commercial suppliers, with authentication being managed by the respective providers. In certain instances, cell lines were generously supplied by established collaborators; while we did not independently verify their authentication, these cells have been used in several peer-reviewed publications and were used only for confirmatory cross-validation experiments. SupT1, Jurkat Flp-In and Jurkat Flp-In T-REx were cultured in RPMI-1640 with 1 mM sodium pyruvate and 10mM HEPES, while HEK293 Flp-In T-REx, HeLa, HEK293T and Huh-7 were cultured in DMEM. Cell culture media was supplemented with 10% fetal bovine serum (FBS), 1x penicillin/streptomycin (Sigma Aldrich, #P4458), 1.25 μg/mL amphotericin B (Sigma Aldrich, #A2942) and the following specific antibiotics: 100 μg/mL Zeocin (Thermo Fisher Scientific, #R25001) for Jurkat Flp-In; 100 μg/mL Zeocin and 7.5 μg/mL Blasticidin S Hydrochloride (Cambridge Bioscience, #B001-100mg) for Jurkat Flp-In T-REx; 350 μg/mL Hygromycin B (Cambridge Bioscience, #H011-20mL) and 7.5 μg/mL Blasticidin for inducible Jurkat Flp-In T-REx EGFP, PURA-EGFP and PURB-EGFP; and 100 μg/mL Zeocin and 5 μg/mL Blasticidin for HEK293 Flp-In TRex. All cells were cultured in a humidified incubator at 37°C with 5% CO_2_. Only mycoplasma-negative cells, confirmed by regular testing, were used in this study.

Full length, infectious HIV-1_mCherry-Nef_ was obtained by transfection of pNL4.3-mCherry-T2A-Nef plasmid into HEK293T. Single round viruses HIV-1_R-E-mCherry-Nef_ (mCherry tagged to Nef) and HIV-1_R-E-Gag-mCherry_ (mCherry tagged to matrix) were produced by co-transfecting HEK293T cells with pNL4-R-E-mCherry-Nef or pNL4.3-R-E-mCherry-gag plasmids,^62^ a plasmid encoding the vesicular stomatitis virus glycoprotein (pHEF-VSVG, NIH AIDS Reagent Program, #4693) and, in the case of HIV-1_Gag-mCherry_, also pPAX-2 (Addgene plasmid #12260).

### Method details

#### Plasmids and recombinant DNA procedures

To generate HIV-1_mCherry-Nef_ we followed a strategy analogous to^86^ but using mCherry (pNL4.3-mCherry-T2A-Nef) instead Renilla luciferase. The pcR-Blunt-NL4.3-mCherry-T2A-Nef plasmid, used for *in vitro* transcription of HIV-1 genomic RNA, was generated by cloning the fragment between FspAI and PdiI from pNL4-3.mCherry-T2A-Nef into pcR-Blunt vector (Thermo Fisher Scientific, #K270020).

Rev-tagged plasmids pNL4.3.R-E-mCherry-T2A-Nef-Rev-Flag-3xMyc and pNL4.3.R-E-mCherry-T2A-Nef-Rev-Halo were made as follows. A synthetic DNA sequence containing a BamHI site, 173 bp of the NL4-3 Rev C-terminal domain, a flexible glycine-serine linker (TCGGCCGGAGGA), the relevant tag, a stop codon and a HpaI site was generated and inserted into pNL4.3.R-E-mCherry-T2A-Nef using BamHI and HpaI restriction sites. pNL4.3.R-E-mCherry-T2A-Nef-ΔRev plasmid was made by introducing 2 point mutations (T5974C and T6041A) in the Rev ORF of the pNL4.3.R-E-mCherry-T2A-Nef plasmid. Firstly, pNL4.3.R-E-mCherry-T2A-Nef was treated with BamHI-HF and EcoRI enzymes to generate a 2.7kb template. To generate the T5974C point mutation, fusion PCR was performed with T5974C_Primer_A, T5974C_Primer_D, T5974C_Primer_B and T5974C_Primer_C. This process was then repeated with primers targeting the T6041A point mutation.

Plasmids encoding RBP-tagged proteins for the generation of inducible cell lines were cloned by 1) amplification of the protein with specific primers from SupT1 cDNA or available template plasmids, and 2) insertion into a pcDNA5/FRT/TO-EGFP-linker vector containing the EGFP tag before (N terminus tagging) the multicloning site.^63^ Single-guide (sg)RNA expression plasmids for CRISPR/Cas9-mediated knock out were generated by inserting annealed oligos into the BLADE plasmid as described before.^87^ All plasmids were validated by sequencing.

#### Gene knock-out in SupT1 and HEK293 cells

We produced nanoblades loaded with Cas9 and specific sgRNAs targeting two regions for each gene as described before.^87^ Nanoblades particles loaded with the Cas9 protein and specific sgRNAs targeting the gene of interests were used in SupT1 and HEK293 Flp-In T-Rex cell lines. In both cases, we transduced 1 × 10^5^ cells with 5–20 μL of nanoblades and 4 μg/mL polybrene in 200 μL of growth medium. 4 h later, 300 μL of fresh medium with 10% FBS was added to reach 500 μL. Cells were incubated for 48 h and then assessed for editing efficiency by genomic DNA extraction (Monarch Genomic DNA Purification Kit, New England Biolabs, #T3010S) followed by PCR, PCR cleanup (QIAquick PCR Purification Kit, Qiagen, #28104), sequencing and sequence trace decomposition using TIDE.^85^ Single knock out (KO) clones were obtained by serial dilution and validated by western blot and cytoplasmic mRNA quantification by RT-qPCR. Huh-7 PURA KO cells were a kind gift of Dr. Mariano A Garcia-Blanco.^61^

#### Virus production

To generate infectious HIV-1_mCherry-Nef_, we transfected HEK293T with pNL4.3-mCherry-T2A-Nef plasmid.^88^ The supernatant was collected 48 h post-transfection (hpt), cleared by centrifugation (3000g for 10 min, 4°C), filtrated with a 0.45μm PVDF Stericup-HV system (Merck, #S2HVU01RE), and precipitated with PEG 6000.^88^ This primary virus stock was titrated by infecting SupT1 and counting mCherry-expressing cells in a flow cytometer. To produce the virus in SupT1, we infected cells at MOI 0.1 by spinoculation and expanded the infected culture by replacing the growth medium every 48 h, keeping cell concentration at 1 × 10^6^ cells/ml. Viruses were purified as above.

To obtain samples for ‘in virion RNA-interactome capture’ (ivRIC), infected SupT1 cells were mixed with uninfected cells at a 1:4 ratio and a concentration of 1 × 10^6^ cells/ml. Medium was replaced by centrifugation at 400*g* for 5 min, and cells were incubated at 37°C and 5% CO_2_ in a T175 flask with 120 mL of medium. At 48 hpi, cells were collected by centrifugation (400g for 5 min) and supernatant was kept. Cell pellet was washed in PBS 1X and lysed in 5 mL of RIC lysis buffer 1X (20 mM Tris-HCl pH 7.5, 0.5 M LiCl, 1 mM EDTA, 0.1% IGEPAL, fresh 0.5% LiDS wt/vol and fresh 5 mM DTT) during 1h at 4°C. Cell lysates were homogenized by pipetting and passing them through a 27G needle several times, and frozen at −80°C for whole cell proteome analysis. Supernatants were further cleared by centrifugation (3000g for 10 min at 4°C), followed by filtration with 0.45μm PVDF Stericup-HV filters. Viral particles were purified on a 10% sucrose cushion (50 mM TrisHCl pH 7.4; 100 mM NaCl; 0.5 mM EDTA; 10% sucrose) at a 4:1 vol:vol ratio in 50 mL conical tubes (Sarstedt, #62.546.254) by centrifugation at 10000*g* for 4h and 4°C.^89^ The pellet was resuspended in PBS 1X containing 50 mM HEPES, incubated overnight at 4°C, and finally aliquoted and stored at −80°C until use.

Single-round, pseudotyped HIV-1 particles were produced by co-transfecting HEK293T cells with pNL4-3.R-E− derived plasmids together with pHEF-VSV-G using calcium phosphate. Virus was collected and concentrated 48 hpt^88^ and titrated by flow cytometry.

For analysis of HIV-1 reverse transcription in viruses produced in WT or PURA KO cells, and second round of infection studies, HEK293 Flp-In T-Rex WT and PURA KO were co-transfected with pNL4-3.R-E-mCherry-T2A-Nef and pHEF-VSVG plasmids as described above. Virus-containing supernatants were collected 48 hpt, cleared by centrifugation (2000g for 10 min, 4°C), filtrated with a 0.45μm PVDF Stericup-HV, and viral particles were purified on a 10% sucrose cushion (50mM TrisHCl pH7.4; 100mM NaCl; 0.5mM EDTA, 10% Sucrose) at 1:3 (sucrose:supernatant) in 50mL flacon tube by centrifugation at 10000*g* overnight at 4°C and resuspended in PBS 1X, 50mM HEPES, pH 7.4.

#### Virus titration by RT-qPCR

The number of viral particles employed in ivRIC was calculated as follows. 100 μL of purified HIV-1_mCherry-Nef_ (or uninfected samples as control) were mixed with 100 μL of RIC lysis buffer 2X (40 mM Tris-HCl pH 7.5, 1 M LiCl, 2 mM EDTA, 0.2% IGEPAL, fresh 1% LiDS wt/vol and fresh 10 mM DTT) and incubated for 1h at 4°C to inactivate the virus. Poly(A) RNA was purified using 100 μL of oligo(dT)_25_ beads (New England Biolabs, #S1419S) following the procedure described in the ‘ivRIC’ section, but eluting in 100 μL. Eluted RNA concentration was measured in a Qubit 2.0 Fluorometer using Qubit RNA High Sensitivity Assay Kit (Thermo Fisher Scientific, #Q32855), and HIV-1 RNA at different sample dilution was quantified using the Luna Universal One-step RT-qPCR kit (New England Biolabs, #E3005S) with specific primers against the 5′ end of HIV-1 gRNA (Key Resources Table). RT-qPCRs were performed on a CFX96 Touch Real-Time PCR Detection System (Bio-Rad).

HIV-1 RNA abundance was calculated in reference to a standard curve (Ct value vs. RNA copy number) generated with purified, *in vitro* synthesized HIV-1_mCherry-Nef_ RNA. This RNA was generated from 1 μg of pcR-Blunt-NL4.3-mCherry-T2A-Nef plasmid that was the template to synthesize capped HIV-1 RNA with the HiScribe T7 ARCA mRNA Kit with tailing (New England Biolabs, #E2060S). The reaction was incubated for 2 h at 37°C in 20 μL. The template DNA was removed by treatment with 4 units of DNase I for 15 min at 37°C. Poly(A) tail was added with E. coli Poly(A) Polymerase for 30 min at 37°C. The *in vitro* synthesized HIV-1 RNA was purified using oligo(dT)25 beads and quantified in a Qubit 2.0 Fluorometer as above. Then, 5 × 10-fold serial dilutions were prepared for the One-step RT-qPCR reaction described above to obtain the corresponding Ct values and generate a standard curve. HIV-1 RNA copy number in ivRIC eluates was estimated by interpolating in the standard curve, using the formula:

[1 × 10^−9^ g/ng ^∗^ 6.022 ^∗^ 10^23^ molecules/mol ^∗^ amount of ssRNA in ng]/[(An x 329.2) + (Un x 306.2) + (Cn x 305.2) + (Gn x 345.2) + 159 g/mol],^90^ similar to the commercial kit Lenti-X qRT-PCR Titration Kit(Takara, #631235).

Data was analyzed with CFX Manager Software v3.1 (Bio-Rad). The number of viral particles was estimated considering that each virion contains 2 copies of viral RNA.

To estimate the percentage of HIV-1 RNA relative to total poly(A) RNA in purified virions (Figure 1E), we measured total RNA concentration using Qubit after oligo(dT)25 RNA isolation from viral particles. The expected RNA copy number if 100% of poly(A) RNA molecules were HIV-1 RNA was estimated using the previous formula and the nucleotide content of HIV-1_mCherry-Nef_ genome. Finally, the real proportion of HIV-1 RNA in purified virions was obtained from RNA copy number measured by RT-qPCR:

%HIV1RNA=MeasuredHIV1RNAcopynumber∗100/ExpectedHIV1RNAcopynumber.

#### HIV-1 reverse transcription analysis

The analysis of HIV-1 reverse transcription (RT) was carried out using a protocol previously described with minor modifications.^91^ Prior to infection, to eliminate free DNA, 50μL of HIV-1 particles generated in WT or PURA-KO cells (HEK293 Flp-In T-Rex or Huh-7) were treated with 4U of TurboDNase I (Invitrogen, #AM2238) plus 4mM of MgCl2 for 1h at 37°C. CA-p24 levels were quantified with the DuoSet ELISA kit (Bio-Techne, #DY7360-05). 0.8 × 10^6^ WT or PURA KO cells (HEK293 Flp-In T-Rex or Huh-7) were seeded in 6-well plates, infected with single round HIV-1_R-E-mCherry-Nef_ virus at MOI 1 upon normalisation by CA-p24 levels, and incubated at 37°C for 2 h in 5% FBS DMEM. Cells were then washed twice with PBS 1X and incubated at 37°C in 5% FBS DMEM. Cells were collected at 0, 2, 4 and 8 hpi in PBS 1X and sedimented at 1500g for 10min. Total DNA was extracted using Monarch Genomic DNA Purification Kit (New England Biolabs). 50 ng of DNA was used for each reaction, along with 100μM of primers (see Key Resources Table) and qPCR Brilliant III SYBR Master Mix (Agilent Technologies Inc, #600883) to a total volume of 10μL per well. The amplification was carried out in a MicroAmp Optical 96-Well Reaction Plate (Applied Biosystems, #N8010560). qPCR data was analyzed using the ΔΔCt method normalised to GAPDH levels. Difference in distribution between two samples were analyzed by unpaired *t* test.

#### RNA sequencing of purified viral particles

The compositional analysis of oligo(dT) isolated RNA from sucrose purified viral particles was done by RNA sequencing as follows. Uninfected cells were mixed with highly infected cells at a 10:1 ratio at a concentration of 1×10^6^ cells/ml and co-cultivated for 48h. Cell pellet was sedimented at 400 rcf and supernatant precleared at 3000 rcf for 10 min before filtering through 0.45μm PES filter. Viral particles were purified over 10% sucrose cushion by centrifugation at 10000 rcf for 4h at 4°C. Total RNA was purified by Trizol extraction and processed with Illumina Truseq stranded kit with Invitrogen ERCC RNA Spike-In Mix and sequenced on an Illumina NextSeq 500 sequencer using a high-output cartridge (Cat# 20024907).

#### Quality control of HIV-1 viral preparations

Viral particles were purified as above. Producer cells were lysed in 1x RIPA buffer (50mM TRIS pH 7.5, 150mM NaCl, 0.1% SDS, 0.5% sodium deoxycholate, 1% Triton X-100) supplemented with 50 μg/ml AEBSF protease inhibitor, while the supernatant was mixed 1:1 with 1x RIPA buffer, and the viral pellet (after sucrose cushion purification) was resuspended in 1x RIPA buffer. Samples were heat inactivated for 1h at 60°C before analysis by SDS-PAGE probed with antibodies specific to PTPRC/CD45 (ThermoFisher, #80297-1-RR100UL), SPN/CD43 (Proteintech, #66224-1-Ig), SERINC3 (Proteintech, #20267-1-AP), p24 (NIBSC – Center for AIDS Reagents, Cat#ARP3279), ACTB (Merck, Cat#A1978) following the protocol outlined below.

#### *In virion* RNA interactome capture (ivRIC)

7 × 10^11^ purified HIV-1_mCherry-Nef_ particles per condition (or the equivalent volume from uninfected cells) were spread on a 6-well plate in 1 mL of total volume (topped up with 1x PBS) on ice and were irradiated with 150 mJ/cm^2^ of UV light at 254 nm. 333 μL of RIC lysis buffer 4X (80 mM Tris-HCl pH 7.5, 2 M LiCl, 4 mM EDTA, 0.4% IGEPAL, fresh 2% LiDS wt/vol and fresh 20 mM DTT) was added to achieve 1X. Samples were recovered using a rubber cell scraper, transferred to a tube and homogenised by pipetting. Lysates were incubated for 1 h at 4°C to ensure the full inactivation of the virus, and then kept at −80°C.

Samples were thawed at room temperature, and 10% of the sample was taken as input (i.e., viral particle proteome). The rest of the sample was incubated under gentle rotation for 2 h at 4°C with 450 μL of oligo(dT)_25_ beads, pre-equilibrated with RIC lysis buffer. Beads were collected in the magnet and the supernatant was stored at 4°C in a new tube. Beads were washed once with 1.5 mL of 1X RIC lysis buffer for 5 min at 4°C, inverting the tubes gently 10 times every minute. Beads were subsequently washed twice with 1.5 mL of cold RIC buffer 1 (20 mM Tris-HCl pH 7.5, 500 mM LiCl, 0.1% LiDS wt/vol, 1 mM EDTA, 0.1% IGEPAL and 5 mM DTT), twice with 1.5 mL of cold RIC buffer 2 (20 mM Tris-HCl pH 7.5, 500 mM LiCl, 1 mM EDTA, 0.01% IGEPAL and 5 mM DTT) and finally twice with 1.5 mL of room temperature RIC buffer 3 (20 mM Tris-HCl pH 7.5, 200 mM LiCl, 1 mM EDTA and 5 mM DTT). Beads were resuspended in 200 μL of elution buffer (20 mM Tris-HCl pH 7.5 and 1 mM EDTA) and incubated for 3 min at 55°C with agitation (200 rpm). Beads were recycled in 0.1M NaOH for 5 min at 55°C with agitation (200rpm), equilibrated in 1X RIC lysis buffer, and re-used for one additional capture following the same protocol. Eluates of the first and second round of oligo(dT) capture were combined and stored at −80°C. Total eluted RNA was quantified using the Qubit RNA High Sensitivity Assay.

For proteomics, samples were treated with RNase A/T1 mix for 1.5 h at 37°C and then 15 min at 50°C as previously described.^92^ Whole cell lysates were treated with 250U/ml of benzonase (Millipore, #70746-4) for 30 min. Protein concentration was measured using Pierce 660m Protein Assay Reagent and Ionic Detergent Compatibility Reagent (Thermo Fisher Scientific, #22660 and #22663 respectively) on a microplate reader (CLARIOstar Plus, BMG Labtech). Samples were further processed by single-pot, solid-phase-enhanced sample preparation (SP3) as described in.^93^ For conventional protein analyses, ivRIC eluates were concentrated on an Amicon Ultra-0.5 centrifugal filter unit 3KDa cut-off (Millipore, #UFC500324) following manufacturer’s recommendations. RNA in eluates was digested with RNases T1 and A as above.

#### Conventional protein analyses

Samples were mixed with NuPAGE LDS Sample Buffer 4X (Thermo Fisher Scientific, #NP0008), incubated for 10 min at 70°C, resolved by SDS-PAGE and analyzed by 1) Western Blot using specific antibodies (Key Resources Table and Table S6), LI-COR Odyssey Fc imaging system for visualization and the Image Studio Software for quantification, or 2) silver staining using the SilverQuest kit (Invitrogen, #LC6070). Statistical significance of Western blot quantifications was analyzed by two-way ANOVA with Dunnett’s correction.

#### HaLo labeling and single molecule RNA FISH

High Precision Coverslips (Marienfeld, #0107052) were washed once in 1 M HCl for 30 min on a rocking machine, twice in double distilled water for 10 min and once in ethanol 70% for 10 min 150,000 HeLa cells were seeded on the prewashed coverslips in wells of a 6-well plate and incubated in DMEM with 10% FBS. Cells were infected 24 h later using VSV-G pseudotyped single round virus. At 48 hpi, cells were washed with PBS and fixed with 4% paraformaldehyde for 10 min at room temperature. Cells were washed with PBS three times with gentle rocking, permeabilised with PBS +0.1% Triton X-(PBSTX), and washed three times with PBSTX for 5 min with gentle rocking. Cells were incubated with 50 nM of Janelia Fluor Halo-646 ligand (Promega, #GA1120) in PBSTX. Coverslips were gently washed 3 times with PBSTX before incubation for 5 min at 37°C with 2 μg/mL of DAPI in PBSTX for 5 min. Cells were then washed twice with PBSTX, once with PBS for 5 min, and once with milliQ H_2_O, followed by mounting on glass slides using Vectashield Antifade mounting medium (Vector Laboratories, #H-1000).

RNA FISH probes were designed using the LGC Biosearch Technologies’ Stellaris RNA FISH Probe Designer focusing on the first 2000 bp of the gag-pol ORF that is only present in gRNA. For single molecule (sm)FISH, cells were seeded and treated as above. After permeabilization, coverslips were washed at 37°C for 10 min with PBSTX, PBSTX with 1X saline sodium citrate solution (SSC), PBSTX with 2X SSC and finally in pre-hybridisation buffer (2x SSC and 10% deionized formamide in DEPC water). Next, cells were incubated in a wet chamber for 16 h at 37°C with 125 nM HIV-1 gRNA-specific Stellaris probes (LGC Biosearch Technologies) in hybridization buffer (2x SSC, 10% deionized formamide and 10% dextran sulfate in DEPC water). Cells were subsequently washed twice with pre-hybridization buffer for 10 min at 37°C and incubated with DAPI and mounted as above.

For the HEK293 PURA KO analyses, 2 × 10^5^ cells were seeded on coverslips (Fisherbrand Glass Circle Coverslips, #12333138) prewashed in 100% ethanol for 30 min and precoated with poly-L-Lysine solution (Sigma-Aldrich, #P4832) for 5 min at 37°C in a 24-well plate, and incubated in DMEM supplemented with 10% FBS. The following day, the cells were infected using MOI 0.5 HIV-1_R-E-Gag-mCherry_ in DMEM with 5% FBS. 24 hpi, cells were washed twice with PBS 1X and fixed as above, with the difference that after the wash with pre-hybridization buffer and PBS, cells were incubated with DAPI (Invitrogen, #D1306) and Cell Mask (Invitrogen, #C37608) with 1:1000 and 1:2000 dilution ratio, respectively, for 30 min at 37°C. After 2 washes with PBS and one with nuclease free water, the coverslips were mounted on glass slides using SlowFade Diamond Antifade Mountant. Images were acquired using a 63x/1.40 oil DIC M27 Plano Apochromat objective lens with a total magnification of 63× on a Zeiss LSM 880 Axio-Observer confocal microscope. Cell and nuclear segmentation was performed in ImageJ using 2D maximum intensity projected images of CellMask Green and DAPI stains. Total number of cells, cytoplasm and nucleus per image was counted in ImageJ. Single-molecule-level quantification of smFISH images was performed using a custom Python pipeline.^68^ Tif files were converted to a numpy array, and individual cells were segmented as described above. Images where cells were labeled with the CellMask stain were pre-processed with a median filter, radius = 50. Background signal in the smFISH channel was subtracted using ImageJ. Threshold setting for smFISH spot detection was set specifically based on mock samples for each set of images collected in each session. ImageJ was also used to manually quantify the intensity of the transcription foci on HIV-1 infected cells. Nucleoplasmic foci with fluorescence intensity evidently superior that single gRNA molecules were considered transcription sites as previously described.^94^ Statistical significance was analyzed by unpaired *t* test with Welch correction where n.s. denotes not significant, ^∗∗∗∗^ denotes *p* < 0.001.

#### Analysis of cell viability and proliferation

To evaluate cell growth and viability, WT and KO cells were seeded at a concentration of 4 × 10^5^ cells/ml. 24, 48 and 72 h later, the number of cells and the percentage of living cells after trypan blue staining was estimated in a Countess II FL Automated Cell Counter (Thermo Fisher Scientific). Cell viability was also assessed by adding CellTiter96 Aqueous One Solution Cell Proliferation Assay (Promega, #G3580) and measuring absorbance at 490 nm on a microplate reader.

#### Flow cytometry analysis of HIV fitness

We infected 2 × 10^6^ SupT1 cells (WT and KO) with infectious HIV-1_mCherry-Nef_ at 0.1 MOI by spinoculation, replaced growth medium at 2 hpi and incubated the cells for 48 h. Cells were collected by centrifugation at 400*g* for 5 min 1) For viral gene expression assessment, cell pellet was resuspended in 2 mL of fresh medium (approximately 1 × 10^6^ cells/ml). 500 μL of infected cells were fixed in formaldehyde (4% final concentration), incubated 1 h at 4°C and analyzed by flow cytometry. 2) The supernatant of these cells was collected and further cleared by centrifugation at 18000*g* for 10 min. Viral particles were precipitated with PEG 6000^88^ and titrated by RT-qPCR. 3) The remaining viral particle sample was normalised by RNA levels and used to infect 1.5 × 10^5^ SupT1 WT cells (second round) by spinoculation. After 48 h, cells were fixed and analyzed by flow cytometer as before. Flow cytrometry data was analyzed using FlowJo Software (BD Life Sciences). Statistical significance was analyzed by ordinary one-way ANOVA with Dunnett’s correction.

Flow cytometry analysis of HIV-1 infection in HEK293 and Huh-7 cells was done as follows. Cells were seeded at a density of 6 × 10^4^ cells/well with 100 μL DMEM 5% FBS without phenol red in a 96-well plate. After 24 h, cells were infected with 1 and 0.5 MOI using CA-p24 normalised single round HIV-1_R-E-mCherry-Nef_ produced in WT or PURA KO cells (see above). After 24h, cells were harvested using TrypLE Express Enzyme(1X) (Gibco, #12604013) and fixed in 2% PFA for 10 min. Cells were analyzed for red fluorescence using a Guava EasyCyte flow cytometer (Cytek Biosciences) counting 15,000 events. Unpaired *t* test was carried out to analyze statistical significance.

#### Generation of Tet-on inducible Jurkat cells

To obtain the Jurkat Flp-In T-REx cell line, we first linearized pcDNA6/TR plasmid (Thermo Fisher Scientific, #V102520) with SapI restriction enzyme and transfected it into Jurkat Flp-In cells (Thermo Fisher, #R76207) using a Lonza Amaxa Nucleofector II and the Cell Line Nucleofector Kit V (Lonza, #VCA-1003), according to the manufacturer’s recommendations for Jurkat E6-1. Stably transfected single clones were selected by serial dilution in Zeocin plus Blasticidin containing media. Expression of Tet-R repressor was verified by Western blot and tested for inducible expression upon nucleofection of a control EGFP plasmid. To generate PURA/B-tagged inducible expression cell lines, we co-transfected Jurkat Flp-In T-REx cells with pOG44 (Thermo Fisher Scientific, #V600520) and the corresponding pcDNA5/FRT/TO plasmid (Table S6) at a 2:8 ratio. At 48 hpt, zeocin was replaced by Hygromycin B for selection.

#### Protein-protein interaction (PPI) analysis

1.5 × 10^7^ SupT1 cells were seeded in 15 cm dishes and infected with HIV-1_R-E-mCherry-Nef_ and HIV-1_R-E-Rev-FLAG-Myc_ at MOI 1. Cells were pelleted at 48h by centrifugation at 400*g* for 5 min and resuspended in 1 mL ice-cold lysis buffer (50 mM Tris pH 7.5, 150 mM NaCl, 1% Triton X-, 0.5 mM EDTA, 25 U/ml of benzonase, 0.1 mM AEBSF) and lysed for 30 min on ice. Lysates were then vortexed and centrifuged at 300g for 3 min. The supernatant was stored in 1.5 mL Eppendorf tubes. Samples were precleared with 100 μL of Pierce Control Agarose beads (Thermo Fisher Scientific, #26150) for 30 min at 4°C. Beads were collected at 2500g for 2 min and supernatant was then mixed with 40 μL of pre-equilibrated anti-FLAG M2 magnetic beads (Merck, #M8823-1ML) in a new tube and incubated for 1h. Beads were washed 6 times with 1 mL of wash buffer (50 mM Tris pH 7.5, 150 mM NaCl, 0.2% IGEPAL, 0.5 mM EDTA) using a magnet. For elution, beads were resuspended in a mixture of 20 μL of wash buffer and 10 μL 3X FLAG peptide (Merck, #F4799-4MG) and kept for 1 h on ice. The supernatant containing Rev-Flag-Myc was collected and stored. Elution was repeated twice.

Jurkat Flp-In T-REx PURA-EGFP and PURB-EGFP cells were induced with 1 μg/mL of doxycycline overnight. Cells were then infected with 1 MOI of VSV-G-pseudotyped HIV-1_R-E-mCherry-Nef_ for 48 h and then lysed (10 mM Tris HCl pH 7.5, 150 mM NaCl, 0.5mM EDTA, 1% Triton X-100, 2mM MgCl2, 1mM DTT, 0.2 mM AEBSF and 25 U benzonase). Cell lysates were cleared by centrifugation (17000 g, 10 min, 4°C). Lysates were pre-cleared with 40 μl of control agarose beads as above. Supernatants were transferred to a new tube and then incubated with 40 μL of pre-equilibrated GFP_Trap agarose bead slurry (ChromoTek, #gta) for 2 h at 4°C with gentle rotation. Beads were sedimented by centrifugation at 2500*g* for 1 min at 4°C and washed six times with 500 μL of wash buffer (10 mM Tris HCl pH 7.5, 150 mM NaCl, 0.5 mM EDTA, 0.2% IGEPAL, 0.1 mM AEBSF serine protease inhibitor, 1mM DTT). Two additional wash steps were performed without IGEPAL. Proteins were eluted with 50 μL of 200 mM glycine pH 2.5 for 60 s followed by neutralisation with 5 μL of 1 M Tris base pH 10.4. Elution was repeated twice, and eluates were combined.

#### PURA/B binding sites on target RNAs by iCLIP2

PURA and PURB binding sites on target RNAs was determined by iCLIP2. The original iCLIP2 protocol^47^ was used with the following modifications. Jurkat Flp-In T-REx PURA-EGFP and PURB-EGFP cells were induced with 1 μg/mL of doxycycline overnight. Cells were then infected with 1 MOI of VSV-G-pseudotyped HIV-1_R-E-mCherry-Nef_ for 48 h. Next, cells were washed twice in PBS 1X, resuspended in 3 mL of PBS 1X, cross-linked with two rounds of 0.15 J/cm^2^ UV light irradiation at 254 nm, washed with PBS 1X and lysed in 1 mL RIPA buffer (50 mM Tris pH 7.5, 150 mM NaCl, 1% Triton X-100, 0.1% SDS, 0.5% wt/vol Na deoxycholate and 0.2 mM AEBSF). Lysates were incubated for 30 min on ice and then stored at −80°C until use. Lysates were thawed on ice, sonicated with 3 cycles of 10 s at 4°C (with 15 s pause between pulses) using a Digenonde bioruptor at level M, homogenized by passing through a 27G needle several times, and finally cleared by centrifugation (17000 g for 10 min at 4°C). EGFP signal was measured on a microplate reader to normalize the amount of lysate for each sample.

4 U TurboDNase and 5 U RNase I (Thermo Fisher Scientific, #AM2294) were added, mixed with a vortex and incubated for 3 min at 37°C at 1100 rpm. 200 U RiboLock RNase Inhibitor (Thermo Fisher Scientific, #EO0381) was then added, followed by incubation for 3 min on ice. Lysates were pre-cleared with 25 μl of pre-equilibrated control agarose beads for 30 min at 4°C, followed by centrifugation at 2500*g* for 2 min. Supernatants were transferred to a new tube and 2 × 10 μL of sample was taken for size-matched input (SMI) processing. Lysates were incubated with 25 μL of pre-equilibrated GFP_Trap agarose bead slurry for 2 h at 4°C with rotation. Beads were washed twice with 900 μL of cold high-salt wash buffer (50 mM Tris-HCl pH 7.4, 1 M NaCl, 1mM EDTA, 1% IGEPAL, 0.1% SDS, 0.5% sodium deoxycholate and 0.2 mM AEBSF), twice with 900 μL of cold medium-salt wash (20 mM Tris HCl pH 7.4, 250 mM NaCl, 0.05% IGEPAL, 1 mM MgCl_2_ and 0.2 mM AEBSF), and twice with 900 μL of cold PNK wash buffer (20 mM Tris HCl pH 7.4, 10 mM MgCl_2_ and 0.2% Tween 20). RNA 3′-end was dephosphorylated at 37°C for 40 min with agitation in PNK buffer (70 mM Tris HCl pH 6.5, 10 mM MgCl_2_ and 1 mM DTT) with 5 U PNK (New England Biolabs, #M0201L), 0.25 U FastAP alkaline phosphatase (Thermo Fisher Scientific, #EF0654), 0.5 U TurboDNase and 20 U RNasin (Promega, #N2111). Beads were washed once with 500 μL cold PNK wash buffer, twice with the same volume of cold high-salt wash buffer and twice with cold PNK wash buffer. 125 nM of L3-IR-App adapter^95^ was ligated using 30 U T4 RNA ligase I High Concentration (New England Biolabs, #M0437M), 20 U RNasin, 4 U PNK, 22.5% PEG8000 and 5% DMSO in ligation buffer (50 mM Tris HCl pH 7.8, 10 mM MgCl_2_ and 1 mM DTT) at 16°C overnight with shaking at 1100 rpm in the dark. Beads were washed once with 500 μL PNK wash buffer, twice with the same volume of high-salt wash buffer and twice with PNK wash buffer. IP samples and inputs were denatured in 1X NuPage LDS Sample Buffer (Thermo Fisher Scientific, #NP0007) with 100 mM DTT for 5 min at 70°C, spun down for 2 min at 2500 g and separated on a NuPAGE 4–12% Bis-Tris gel (Thermo Fisher Scientific, #NP0321BOX). Protein-RNA complexes were transferred onto an iBLOT2 nitrocellulose membrane (Thermo Fisher Scientific, #IB23001) for 2 h at 30 V and visualized. The region corresponding to the RBP-EGFP band and above was cut (for both IP and SMI samples) and digested using ∼350 μg Proteinase K (Roche, #3115828001) in 180 μL PK-SDS solution (10 mM Tris HCl pH 7.4, 100 mM NaCl, 1 mM EDTA and 0.2% SDS) for 60 min at 50°C with shaking (1100 rpm). RNA was purified by adding 1X volume of Phenol:Chloroform:Isoamyl Alcohol pH 6.6–6.9 (Sigma-Aldrich, #P3803), incubating for 10 min at 37°C with shaking (1100 rpm) and phase separation in MaxTract tubes by centrifugation at 16000*g* for 5 min. RNA was cleaned using Zymo RNA Clean & Concentrator-5 (ZYMO Research, #R1013). For SMI library preparation, samples were treated first with 5 U PNK, 0.5 U FastAP and 20 U RNAsin in PNK buffer pH 6.5 for 40 min at 37°C and 1100 rpm. RNA was cleaned up with Dynabeads MyOne Silane (Thermo Fisher Scientific, #37002D). L3-IR-App adapter ligation was performed with 45 U T4 RNA ligase I High Concentration in 1X T4 RNA Ligase Reaction Buffer with 2% DMSO, 27% PEG8000, and 133 nM L3-IR-adapter for 75 min at room temperature, followed by MyOne bead purification. SMI was treated with 25 U 5′ Deadenylase (New England Biolabs, #M0331S) and 15 U RecJf endonuclease (New England Biolabs, #M0264S) in 1X New England Biolabs buffer 2 with 20 U RNAsin, 20% PEG8000 for 1 h at 30°C and then 30 min at 37°C at 1100 rpm, followed by a MyONE clean-up. RNA from IP and SMI samples were reverse transcribed using Superscript IV reverse transcriptase (Thermo Fisher Scientific, #18090010) and hydrolyzed by adding 1.25 μL of 1 M NaOH for 15 min at 85°C before neutralization with 1.25 μL of 1 M HCl. cDNA was purified using MyOne silane beads. L#clip2.0 adapters with barcodes for multiplexing^95^ were ligated to cDNA by mixing first 2 μL of 10 μM adapter with 5 μL of cDNA and 1 μL of DMSO and incubating at 75°C for 2 min before placing on ice. Then, ligation mix (45 U T4 RNA ligase I High Concentration in 1X RNA ligase buffer with 1 mM ATP and 22.5% PEG8000) was added to the cDNA-bead solution and incubated overnight at 20°C with shaking 1100 rpm. cDNA was cleaned up with MyONE beads before PCR amplification. Pre-amplification was performed using 2X Phusion HF PCR Master mix (New England Biolabs, #M0531L) with P5Solexa_s and P3Solexa_s primers for six cycles, followed by ProNex (Promega, #NG2001) size-selective purification. Optimal qPCR cycles were determined on a CFX96 Touch Real-Time PCR Detection System (Bio-Rad) using EvaGreen (Biotium, #31000), 2X Phusion HF PCR Master mix and P5/P3 Solexa primers. Final PCR products were purified using two consecutive rounds of ProNex Size selection. Libraries were quantified by qPCR using the KAPA Library Quantification DNA standards (Roche, #07960387001) and High Sensitivity DNA kit (Agilent, #5067-4626) in an Agilent 2100 Bioanalyzer instrument. Each group of samples was pooled equimolarly and then mixed at the following proportions: 50% IP library pool, 37.5% SMI library pool, and 12.5% negative control EGFP. Sequencing was performed on a NextSeq 550 sequencer with a 75 cycle High-output kit v2.5 (Illumina, #20024906).

#### PURA - HIV-1 gRNA interaction analysis

1 × 10^7^ HEK293 Flp-In T-Rex EGFP or PURA-EGFP cells were induced with 1μg/ml Doxycycline for 24 h before transfection with pNL4.3-R-E-mCherry-T2A-Nef and pHEF-VSV-G using X-tremeGENE 9 (Merck, #6365779001). 48 hpt supernatant was precleared at 5000rpm for 5 min before filtering through 0.4μm PEF sterile filter. Virus was precipitated by PEG purification and resuspended in 25mM HEPES in D-PBS. Purified virus was lysed in ice-cold RIPA (50mM Tris pH 7.5, 150 mM NaCl, 0.1% SDS, 0.5% sodium deoxycholate, 1% Triton X-100) supplemented with 50μg/ml AEBSF, 2U/ml TurboDNase, 40U/ml RiboLock and 5mM DTT. Immunoprecipitation was performed with 20μL GFP-TRAP agarose/sample at 4°C for 1h. Samples were washed 3x with ice-cold RIPA buffer supplemented with 5mM DTT and 40U/ml Ribolock. RNA from input and beads were isolated using Trizol following manufacturers’ recommendations. RT-qPCR was performed following manufacturers’ recommendations with NEB Luna Universal One-Step RT-qPCR Kit using HIV-1 genomic primers on a QuantStudio3 Real-Time PCR system. Data was normalised to input and visualised as fold change in R using ggplot2.

#### Sample preparation for LC-MS/MS

ivRIC and PURA-EGFP and PURB-EGFP IP eluates were processed by single-pot, solid-phase-enhanced sample preparation (SP3).^93^ Eluates of Rev IP were processed using Filter-Aided Sample Preparation (FASP) protocol.^96^ Peptides were analyzed in an Ultimate 3000 ultra-HPLC system (Thermo Fisher Scientific) as described before.^92^ Protein identification and quantification were performed using Andromeda search engine implemented in MaxQuant (1.6.3.4). Mass spectra were searched against human proteome reference (Uniprot_id: UP000005640, downloaded Nov 2016) and a custom HIV-1 (NL4-3) proteome including all known viral proteins. Flag-Myc-tagged Rev and EGFP were also included in the searches of the Rev and PURA/B interactomes, respectively. Search parameters included: full tryptic specificity with maximal two missed cleavage sites, carbamidomethyl (C) set as fixed modification, acetylation (protein N-term) and oxidation (M) set as variable modifications. False discovery rate (FDR) cut-off for peptide identification was set to 1%. For ivRIC and SupT1 whole cell proteome iBAQ and LFQ options were toggled ON. All other settings were set to default.

#### Mapping and comparing gene IDs

For all datasets analyses, including mass spectrometry data, genes were mapped to Hugo gene nomenclature committee IDs (HGNC) using R package BiomaRt. Any genes that could not be mapped were manually curated by manual addition or were removed if it was a pseudogene or its HGNC entry was missing. For HIV-1 analysis, the HIV-1 NCBI database was downloaded from the NCBI web server and parsed to gene IDs following the same strategy. Upset plots and Euler diagrams were generated using the R packages upsetR and venneuler respectively.

#### GO and STRING network analyses

General GO terms were extracted using the tool EnrichR^83^ (https://maayanlab.cloud/Enrichr/) and summarized using REVIGO.^84^

To generate virus GO term plots, the R package AnnotationDbi was queried with HGNC IDs and resulting GO terms filtered by the following GO categories: ‘viral’, ‘immune’, ‘infection, ’pathogen, ‘immune cell’ and ‘immune molecule’.

Network analyses were performed using the Cytoscape 3.9.1 platform^78^ with the following add-ons: stringApp^79^ for STRING protein network analysis and GO enrichment; clusterMaker2^80^ for clustering data using MCODE algorithm^81^ and DyNet^82^ for comparison of two networks.

We classified proteins as RBPs if they were identified in at least 3 independent RNA interactome studies based on the EMBL RBPbase (https://rbpbase.shiny.embl.de/) resource.

### Quantification and statistical analyses

Statistical analyses and data presentation were performed with Prism v.10 (GraphPad Software) and specific R (The R Foundation) packages as described in the different sections of STAR Methods. Statistical details can be found in figure legends and figures, including the value of n (biological replicates; at least three), means and error bars (standard deviation of all the replicates). Functional data (Figures 4 and S7) was subjected to unpaired *t* test with Welch correction, or one-way or two-way ANOVA followed by Dunnett’s correction, as appropriate; differences were considered significant if *p* < 0.05. Proteomics and iCLIP2 data was analyzed as indicated below.

#### Quantitative analysis of proteomics data

The proteinGroup files of MaxQuant search results were imported in RStudio (R Project) for further processing. Protein intensities were log 2 transformed. In each dataset proteins with less than 2 valid intensity measurements across experimental conditions were removed prior to downstream analysis. Batch effects in each comparison were assessed using principal component analysis (PCA). Normalisation was performed to ivRIC inputs ad PPI analyses using variance stabilisation normalisation (vsn) method.^97^ Missing values were imputed with deterministic minimum method (R package version 2.0. https://CRAN.R-project.org/package=imputeLCMD) using 1% quantile of global intensities. All missing values were imputed in ivRIC and Rev interactome data. For PURA/PURB interactome data, only proteins with all values missing in one condition were imputed as described before.^98^ Linear modeling and Bayesian-model-based moderated *t* test was performed using the R-package *limma*.^69^ Batch effects in ivRIC eluates and Rev and PURA/B interactomes were accounted for by incorporation in modeling using “block” argument provided in *limma*. *p* values obtained in the moderated *t* test were adjusted to account for multiple testing using Benjamini-Hochberg method.

#### iCLIP2 data processing

The raw FASTQ files were demultiplexed according to the sample barcode using Je Suite (Girardot et al., 2016) and adapter trimmed with Cutadapt (Martin, 2011). Trimmed reads were aligned to a concatenated human (GRCh38, ENSEMBL Release 104) and HIV-1 NL4.3 genome using STAR with end-to-end alignment mode.^70^ Only uniquely aligned reads were considered for the downstream analysis. PCR duplicated reads were collapsed using unique molecular identifiers (UMIs) attached to the read header with the Je Suite. The GRCh38 and HIV-1 genomic annotations were pre-processed to generate sliding windows (50nt window, 20nt step size) using HTSeq-clip.^72^ Cross-link truncation sites (position −1 relative to the 5′ end of the read start) were extracted using BEDTools^73^ and quantified against the sliding windows using HTSeq-clip. For peak calling, a R/Bioconductor package DEW-Seq was used to identify significantly enriched sliding windows in PURA/PURB immunoprecipitated samples over the corresponding size-matched input control samples (log2FoldChange >2 and p.adj <0.01).^72^ The Independent Hypothesis Weighting (IHW) method was used for the multiple hypothesis correction.^99^ To remove background signal resulting from non-specific binding of RNA to GFP, significantly enriched sliding windows (log2FoldChange >2 and p.adj <0.01) from GFP-immunoprecipitated control samples were removed. Overlapping significant sliding windows were merged to binding regions, and these sites were curated to 8nt long binding sites based on peak width and maxima. Binding sites were queried against the genome annotation (ENSEMBL release 104) using the GenomicRanges R package to extract overlaps with genes and transcript features.^74^ The overlap information was used to construct the meta-transcript coverage of binding sites and the heatmap distribution of PURA/PURB binding in the proximity of start and stop codons.

Given the repetitive motifs present in 5′ and 3′ of HIV-1 genome, we used a custom transcriptome to identify binding motifs at the 5′ and 3′ untranslated regions (UTRs). In brief, we superimposed the LTR-1 and LTR-2 sequences flanked by 100bp of protein-coding sequences from HIV-1 open reading frames at both 5′ and 3′ ends. HIV-1 reads were initially filtered using BBMap with kmer = 25 mode (https://sourceforge.net/projects/bbmap/), then mapped to the custom transcriptome using STAR. Peak-calling was performed as described above using DEW-Seq except for using smaller sliding windows (10nt window, 2nt step size) and a more lenient threshold (log2FoldChange >1.5 and p.adj <0.01).

Sequences for motif enrichment analysis were defined for each binding site as a 70-nucleotide region, centered on the peak in BigWig signal. For each binding site, a gene and gene region matched background sequence were defined to allow for differential enrichment. Enrichment analysis was performed using HOMER. Motifs were processed and plotted using the R packages universalmotif and ggseqlogo.

## Supplemental information

Document S1. Figures S1–S10

Table S1. Defining the HIV-1 ivRNP This table reports the proteomic results of the ivRIC analysis focusing on the comparison between the eluates from experimental samples and controls.

Table S2. The viral particle and whole CD4^+^ T cell proteome This table reports the proteomic results of the ivRIC inputs and the whole cells and their comparison between them and with the ivRIC eluates.

Table S3. The Rev protein-protein interactome This table reports the proteomic results of the Rev-Flag-Myc immunoprecipitation and controls using anti-Flag antibody and Flag.

Table S4. The PURA and PURB protein-protein interactome This table reports the proteomic results of the immunoprecipitation in HIV-1 infected and uninfected cells of PURA-EGFP, PURB-EGFP and unfused EGFP using the GFP_Trap.

Table S5. The PURA and PURB iCLIP This table reports the RNA sequencing results of the iCLIP2 analysis of PURA-EGFP and PURB-EGFP in mock and HIV-1 infected cells.

Table S6. Key resources List and details of antibodies, primers and synthesized cDNAs used in this study.

Document S2. Article plus supplemental information
