# Supplementary material for: Incorporation of genome-bound cellular proteins into HIV-1 particles regulates viral infection
Source: Cell Rep. 2026 Apr 7;45(4):117090. doi: 10.1016/j.celrep.2026.117090 (PMC13124994; doi:10.1016/j.celrep.2026.117090)
Supplement: Document S1. Figures S1–S10 [file mmc1.pdf]

Cell Reports, Volume *45*

Supplemental information

Incorporation of genome-bound cellular proteins into HIV-1 particles regulates viral infection

Manuel Garcia-Moreno, Azman Embarc-Buh, Robin Truman, Marko Noerenberg, Louisa Iselin, Honglin Chen, Caroline E. Lenz, Jeffrey Y. Lee, Kate Dicker, Snehith Dyavari Shetty, Natasha Palmalux, Quan Gu, Thibault J.M. Sohier, Aino I. Järvelin, Wael Kamel, Vincenzo Ruscica, Emiliano P. Ricci, Ilan Davis, Shabaz Mohammed, and Alfredo Castello

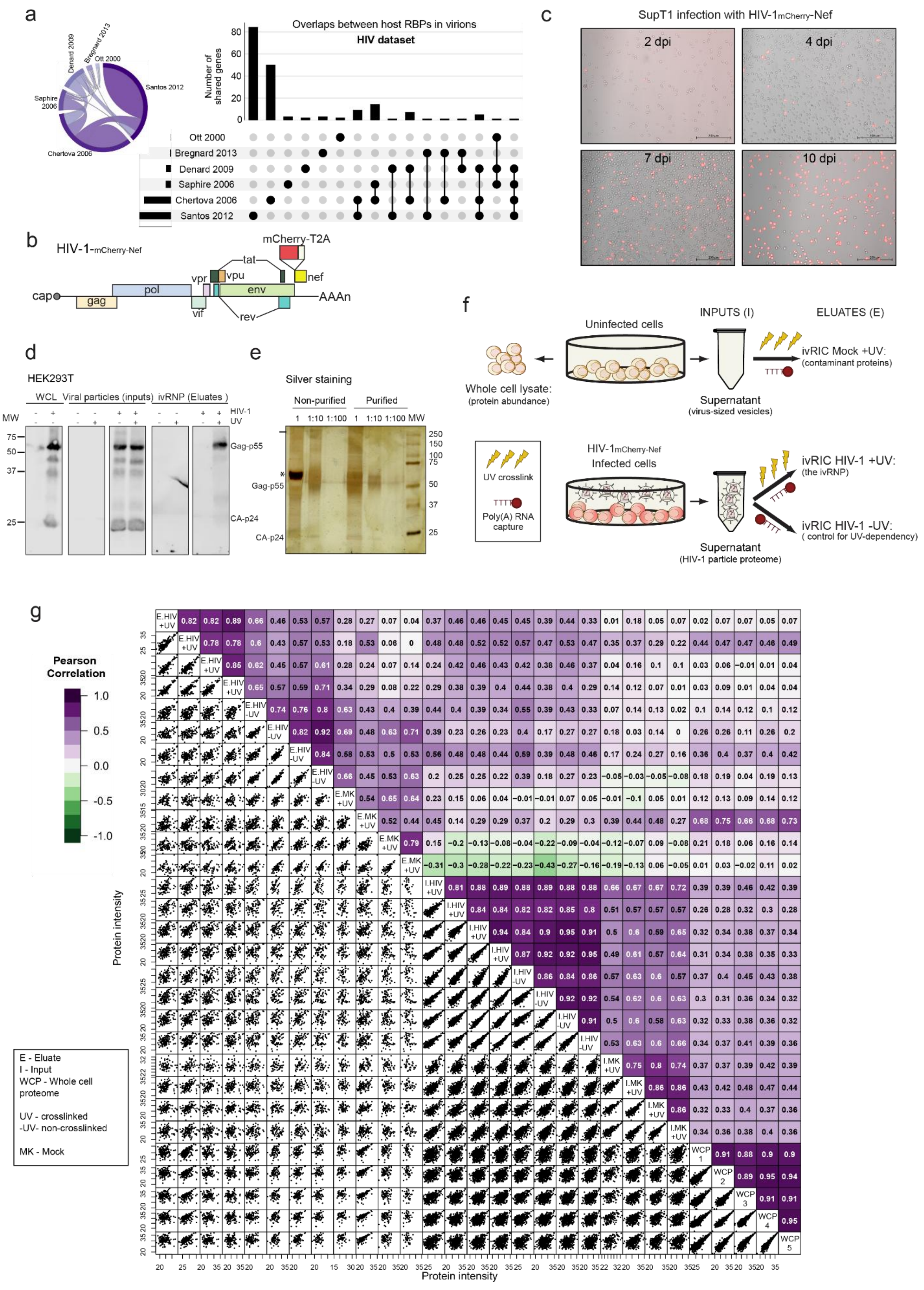
a
Overlaps between host RBPs in virions
HIV dataset
Number of shared genes
Ott 2000
Bregnard 2013
Denard 2009
Saphire 2006
Chertova 2006
Santos 2012
b
HIV-1-mCherry-Nef
mCherry-T2A
cap
gag
pol
vif
vpr
vpu
tat
env
rev
nef
AAAn
c
SupT1 infection with HIV-1mCherry-Nef
2 dpi
4 dpi
7 dpi
10 dpi
d
HEK293T
WCL
Viral particles (inputs)
ivRNP (Eluates)
HIV-1
UV
MW
Gag-p55
CA-p24
e
Silver staining
Non-purified
Purified
1 1:10 1:100
MW
Gag-p55
CA-p24
f
Uninfected cells
INPUTS (I)
ELUATES (E)
Whole cell lysate:
(protein abundance)
Supernatant
(virus-sized vesicles)
ivRIC Mock +UV:
(contaminant proteins)
UV crosslink
Poly(A) RNA capture
HIV-1mCherry-Nef
Infected cells
Supernatant
(HIV-1 particle proteome)
ivRIC HIV-1 +UV:
(the ivRNP)
ivRIC HIV-1 -UV:
(control for UV-dependency)
g
Pearson Correlation
1.0
0.5
0.0
-0.5
-1.0
Protein intensity
E - Eluate
I - Input
WCP - Whole cell proteome
UV - crosslinked
-UV- non-crosslinked
MK - Mock

Supplemental Figure 1 (Figure S1): Proteomic analysis of the ivRNP. a) Comparison of previously established HIV-1 particle proteomes. b) Schematic representation of the HIV-1mCherry-Nef chimeric virus. c) SupT1 cells were infected at MOI 0.1 with HIV-1mCherry-Nef and mCherry-expressing cells were checked by fluorescent microscopy at different days post infection (dpi). d) Western blotting against CA-p24 in whole cell lysate (WCL), input (viral particles) and eluate (vRNP) samples of an ivRIC experiment in HEK293T cells transfected with the plasmid encoding HIV-1mCherry-Nef. e) Silver staining of HEK293T producer cells, their supernatant and after the sucrose cushion purification. * indicates a very prominent serum-derived protein, most likely albumin. f) Schematic representation of the ivRIC experimental design used for proteomic analysis of ivRIC samples. g) Scatter plots showing the protein intensity and the Pearson correlation between different samples and replicates of the ivRIC experiment (n=4). MW, molecular weight. Related to Figure 1.

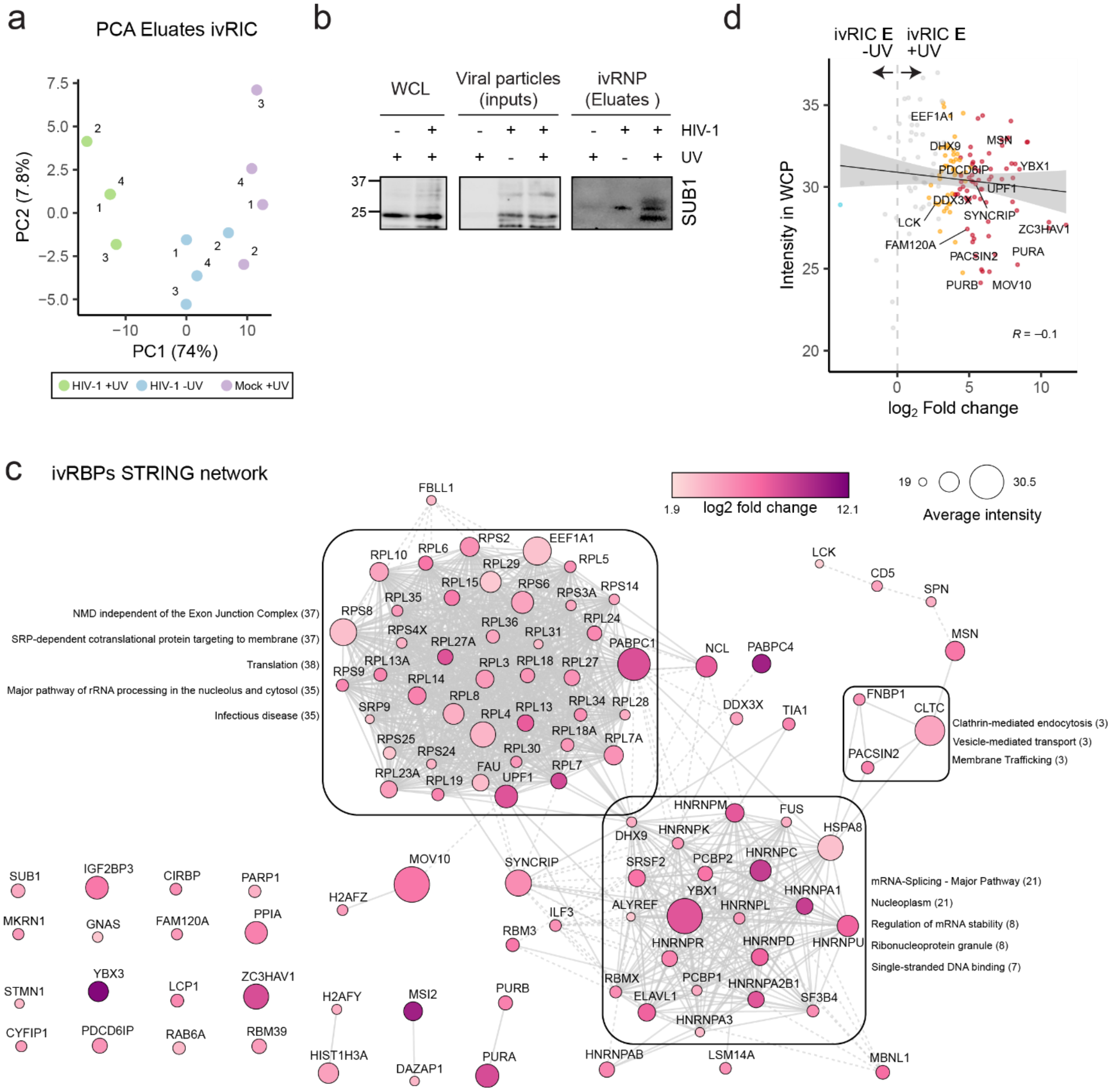

Supplemental Figure 2 (Figure S2): Analysis of the ivRIC proteomic experiment in HIV-1-infected CD4+ T lymphocytic cells. a) Principal component analysis (PCA) of the ivRIC eluates. b) Western blotting analysis of the newly discovered cellular ivRBP SUB1 in whole cell lysates (WCL), inputs and eluates of ivRIC. c) STRING network analysis of the ivRBPs generated by Cytoscape. Physical interactions are represented by solid lines and functional interactions by dashed lines. GO enriched terms are shown for each cluster. Related to Figure 1.

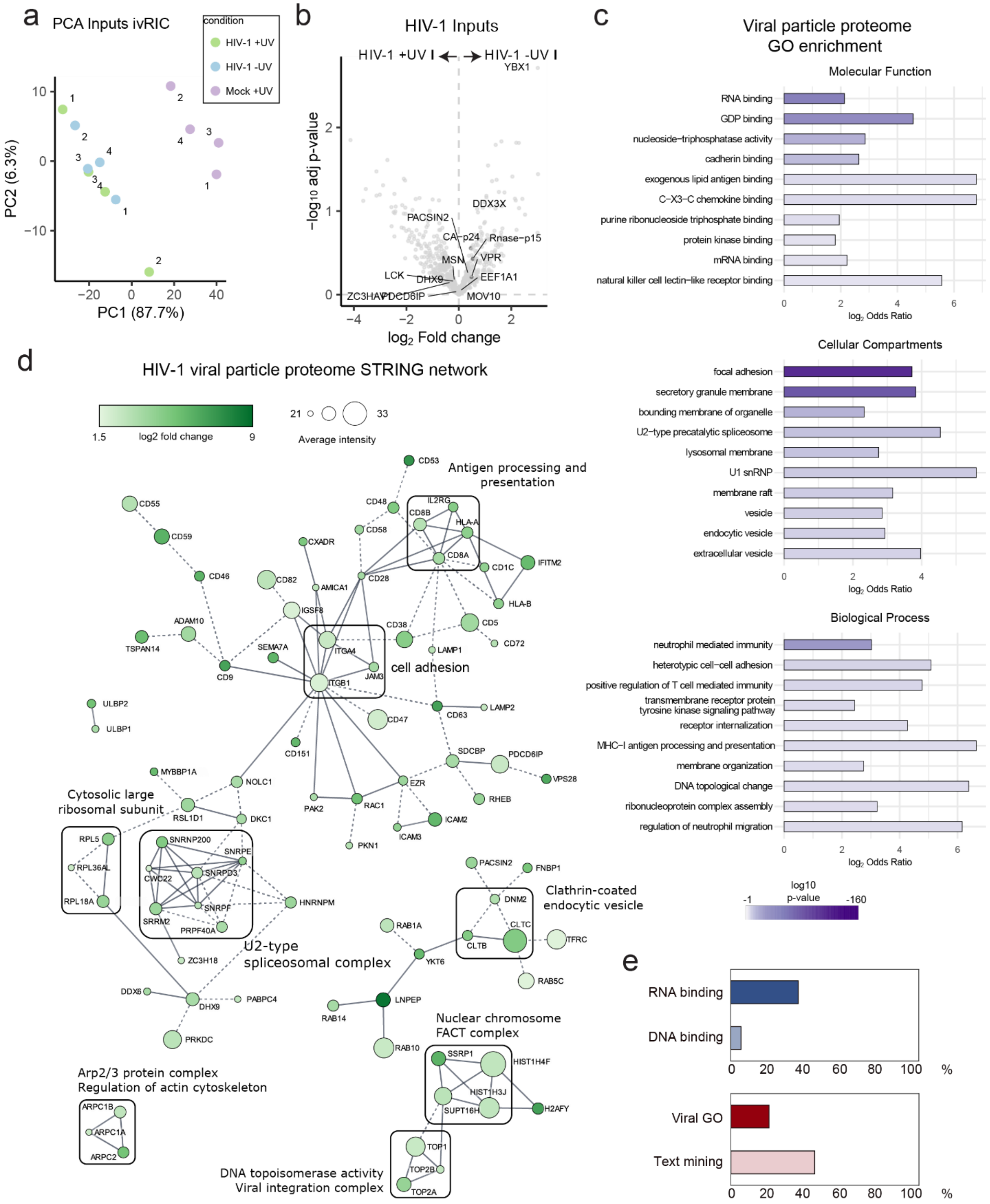

Supplemental Figure 3 (Figure S3): The proteome of HIV-1 viral particles. a) PCA of the ivRIC input samples (purified viral particles). b) Volcano plot comparing the proteome of viral particles (ivRIC inputs) in UV-irradiated and non-irradiated samples. Grey dots are non-significantly enriched proteins. Proteins show no UV dependency

opposite to ivRIC eluates (ivRNP). c) GO term enrichment analysis of the proteins enriched in HIV-1 particles over the supernatant of mock-infected cells. d) STRING network analysis of the proteins enriched in HIV-1 particles using Cytoscape. Physical interactions are represented by solid lines and functional interactions by dashed lines. Top GO enriched terms are shown for each cluster. e) Bar plots showing the proportion of proteins in the viral particles (inputs of ivRIC) annotated with RNA- and DNA-binding (GO terms); virus-related (GO terms) and HIV-1-related (text-mining) functions. Related to Figure 2.

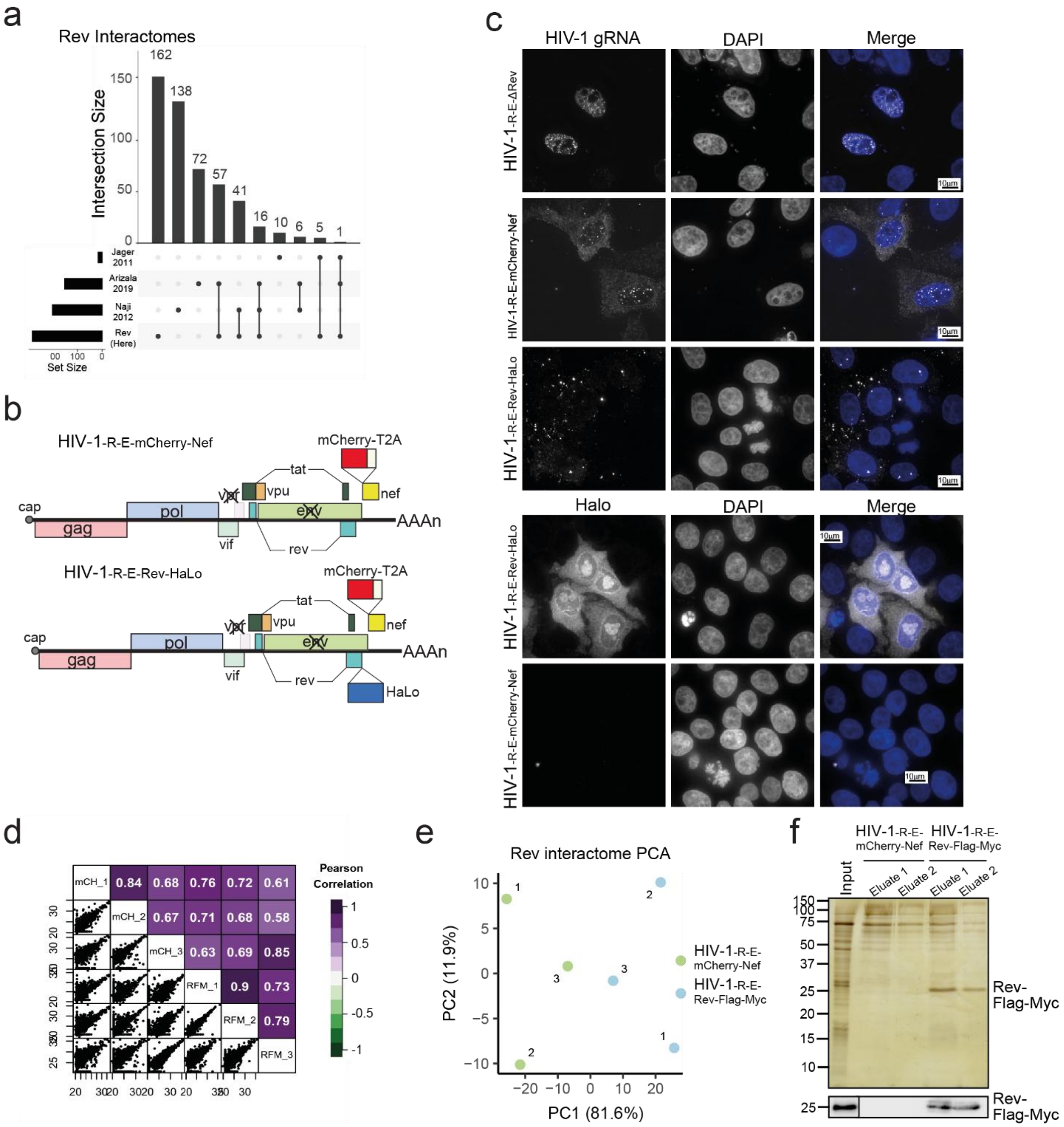

Supplemental Figure 4 (Figure S4): The proteomic analysis of the Rev interactome. a) Overlapping of the previously established Rev interactomes with the dataset generated here. b) Schematic representation of HIV-1R-E-mCherry-Nef and HIV-1R-E-Rev-HaLo. c) HeLa cells infected infected with VSV-G pseudotyped chimeric HIV-1 were observed under a fluorescent microscope and mCherry signal was used as proxy for infection (upper panels). Fluorescence microscopy analysis of Rev-HaLo, and gRNA in cells infected with the chimeric viruses in panel (b) using a confocal microscope (bottom panels). d) Scatter plots showing the protein intensity and the Pearson correlation between different samples and replicates of the Rev protein-protein interaction experiment (n=3). e) PCA of the eluates of the Rev-Flag-Myc IP and the control IPs. g) Silver staining and Western blot of the Rev-Flag-Myc IP. Related to Figure 3.

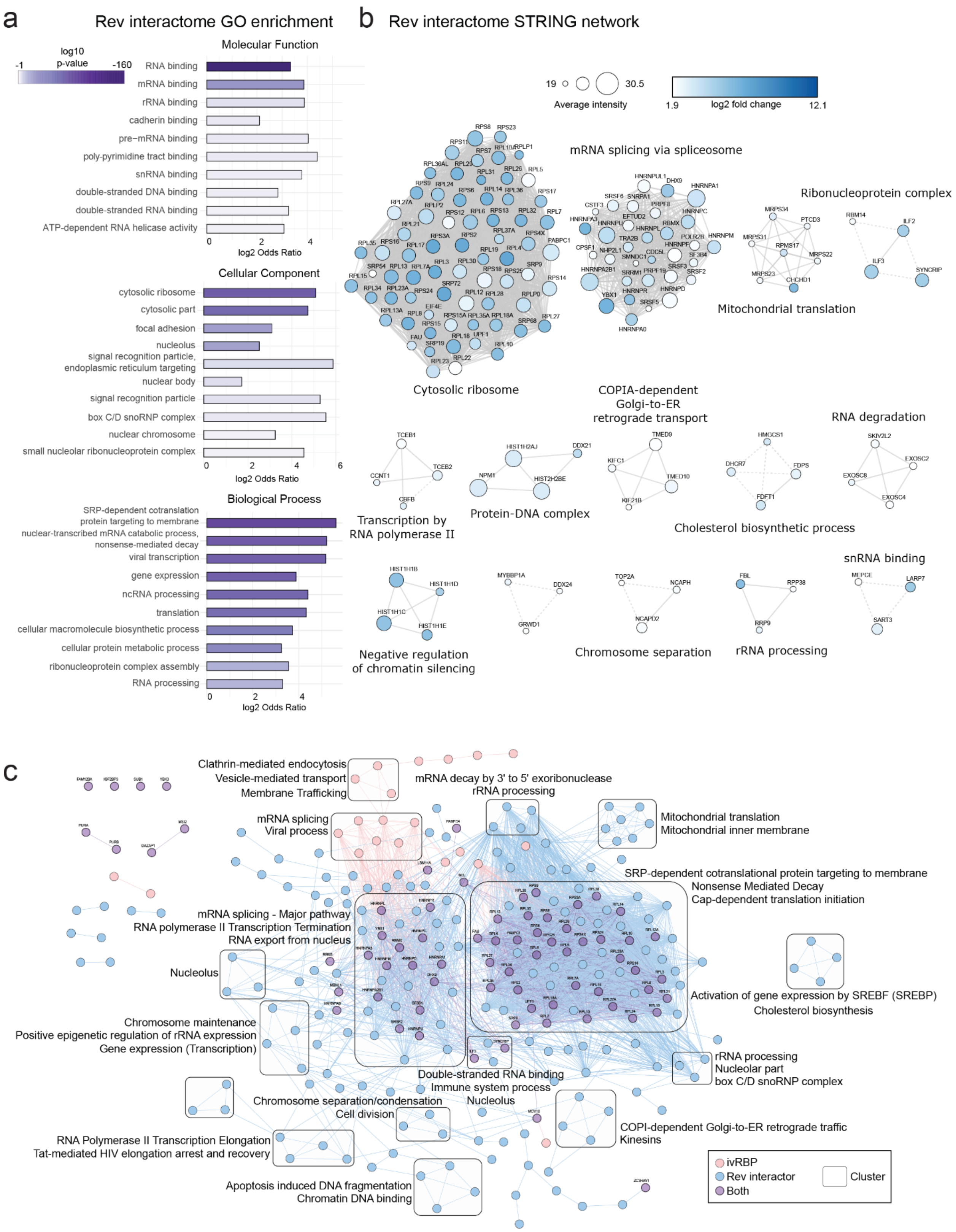

Supplemental Figure 5 (Figure S5): Characterising the ivRNP and the Rev interactome. a) GO enrichment analysis of the Rev interactome. b) STRING clustered

network of the Rev interactome analysed with Cytoscape. Physical interactions are represented by solid lines and functional interactions by dashed lines. Top GO enriched terms are shown for each complex. c) STRING analysis of the proteins identified in the ivRNP and Rev interactomes as in (b). Related to Figure 3.

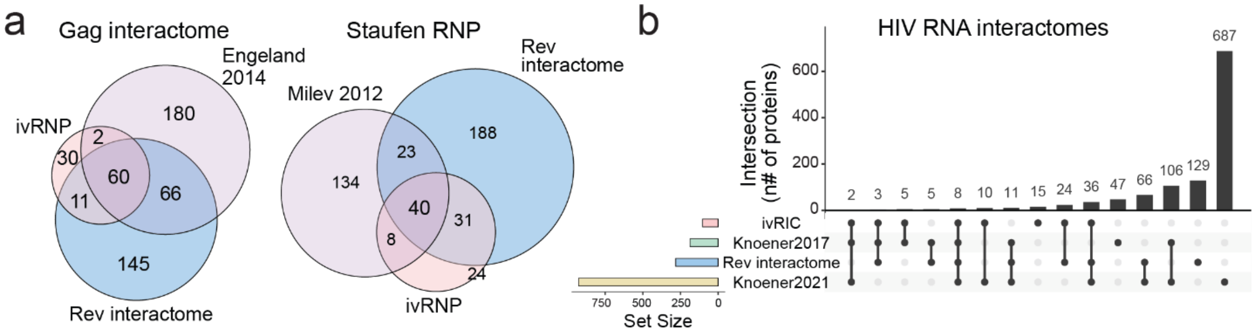

Supplemental Figure 6 (Figure S6): Comparison of the ivRNP and Rev interactome with other relevant datasets. A) Venn diagram showing the overlapping of the ivRNP and the Rev interactome with the previously established Gag41 and Staufen42 interactomes. b) Upset plot comparing the ivRNP and Rev interactome to the previously established HIV-1 RNA interactomes10,43. Related to Figure 3.

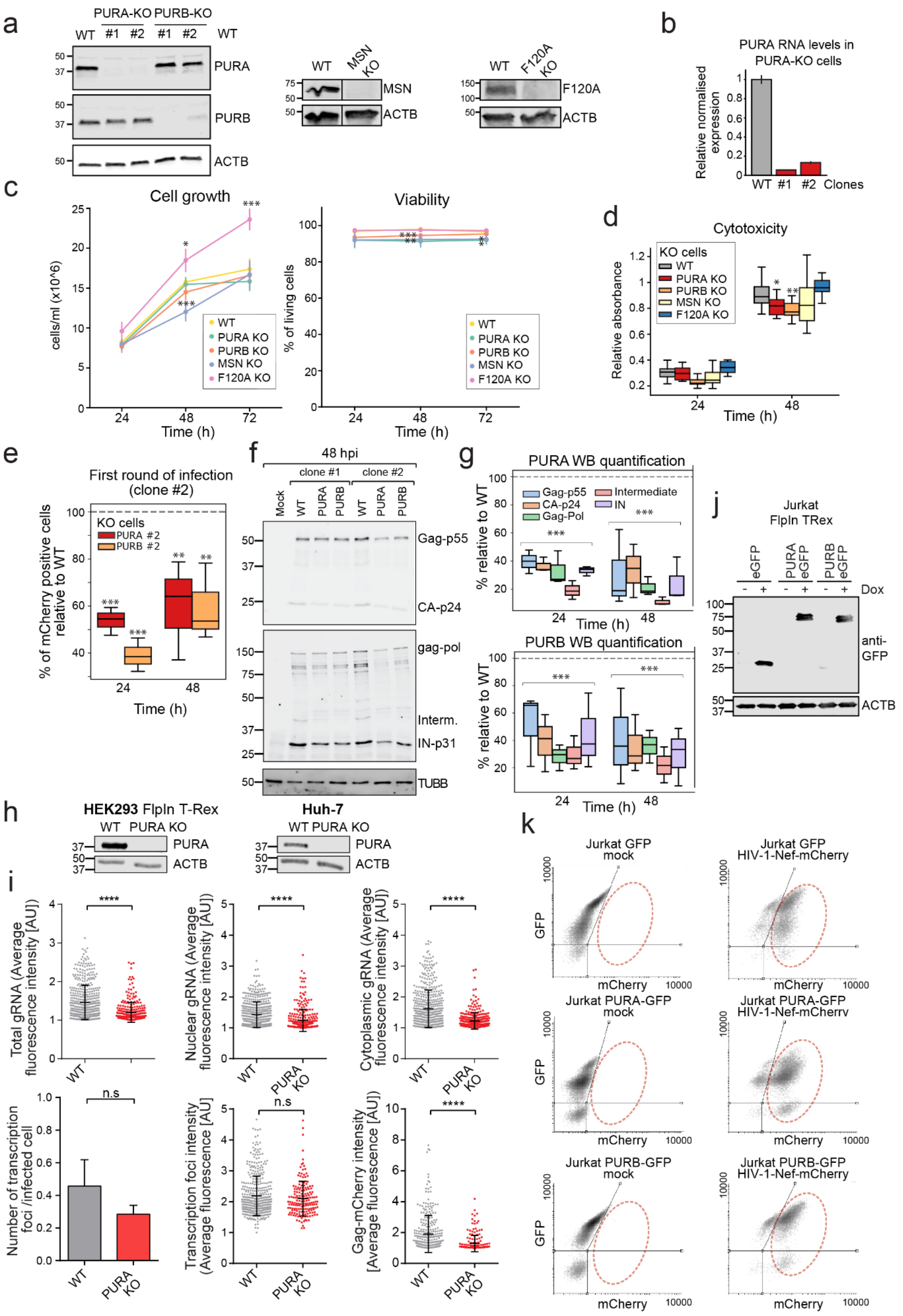
a
PURA-KO PURB-KO
WT #1 #2 #1 #2 WT
PURA
PURB
ACTB
WT MSN KO
MSN
ACTB
WT F120A KO
F120A
ACTB
b
PURA RNA levels in PURA-KO cells
Relative normalised expression
WT #1 #2 Clones
c
Cell growth
cells/ml (x10^6)
Time (h)
WT
PURA KO
PURB KO
MSN KO
F120A KO
Viability
% of living cells
Time (h)
d
Cytotoxicity
Relative absorbance
KO cells
WT
PURA KO
PURB KO
MSN KO
F120A KO
Time (h)
e
First round of infection (clone #2)
% of mCherry positive cells relative to WT
KO cells
PURA #2
PURB #2
Time (h)
f
48 hpi
clone #1
clone #2
Mock
WT
PURA
PURB
Gag-p55
CA-p24
gag-pol
Interm.
IN-p31
TUBB
g
PURA WB quantification
% relative to WT
Gag-p55
CA-p24
Gag-Pol
Intermediate
IN
Time (h)
PURB WB quantification
% relative to WT
Time (h)
h
HEK293 FlpIn T-Rex
WT PURA KO
PURA
ACTB
Huh-7
WT PURA KO
PURA
ACTB
i
Total gRNA (Average fluorescence intensity [AU])
Nuclear gRNA (Average fluorescence intensity [AU])
Cytoplasmic gRNA (Average fluorescence intensity [AU])
Number of transcription foci /infected cell
Transcription foci intensity (Average fluorescence [AU])
Gag-mCherry intensity [Average fluorescence [AU])
WT
PURA KO
n.s
j
Jurkat FlpIn TRex
eGFP
PURA eGFP
PURB eGFP
Dox
anti-GFP
ACTB
k
Jurkat GFP mock
Jurkat GFP HIV-1-Nef-mCherry
Jurkat PURA-GFP mock
Jurkat PURA-GFP HIV-1-Nef-mCherry
Jurkat PURB-GFP mock
Jurkat PURB-GFP HIV-1-Nef-mCherry
GFP
mCherry

Supplemental Figure 7 (Figure S7): Establishment of ivRBP KO cell lines and inducible PURA/B Jurkat cells. a) Western blot analysis of the different KO SupT1 cell lines using specific antibodies. b) RT-qPCR analysis of PURA mRNA in SupT1 PURA KO clones. c) Line plots showing cell proliferation and viability of SupT1 KO cells (n=3). d) Box plot showing cytotoxicity after gene KO (n=3). e) Flow cytometry analysis of mCherry positive cells in SupT1 WT, PURA KO and PURB KO clone 2 infected with HIV-1mCherry-Nef (n=4). f) Western blotting analysis of WT, PURA KO and PURB KO SupT1 cells infected with HIV-1mCherry-Nef for 48hpi. g) Box plots showing the Western blot quantification of different HIV-1 proteins (n=3). For d, e and g: *, p<0.05; **, p<0.01; ***, p<0.001. h) Western blot against PURA in WT and PURA KO HEK293 Flp-In T-Rex and Huh-7 cells. i) Analysis of HIV-1-R-E-Gag-mCherry gene expression using smFISH. The analysis includes whole cell, nuclear, and cytoplasmic gRNA intensity as well as Gag-mCherry signal. We also quantified the number of transcription foci in the nucleoplasm, and the intensity of each individual transcription foci. These analysis were done with n ≥ 250 (gRNA signal) and n ≥ 150 (Gag-mCherry signal) cells per conditions across 3 replicates. ****, p<0.001; n.s., non-significant. j) Western blot showing the doxycycline (dox)-inducible expression of PURA-eGFP and PURB-eGFP in Jurkat Flp-In T-Rex. k) Flow cytometry analysis of mock and HIV-1R-E-mCherry-Nef infected Jurkat cells expressing eGFP-fused proteins at 48 hpi. mCherry positive cells are indicated with a dotted line. Panels B, C and I show the median as a horizontal line and the (+/-) standard deviation as error bars. Panels D, E, and G show box-and-whisker plots, in which the boxes represent the interquartile range (Q1-Q3) with the median indicated, and the whiskers denote the minimum and maximum values. Related to Figure 4.

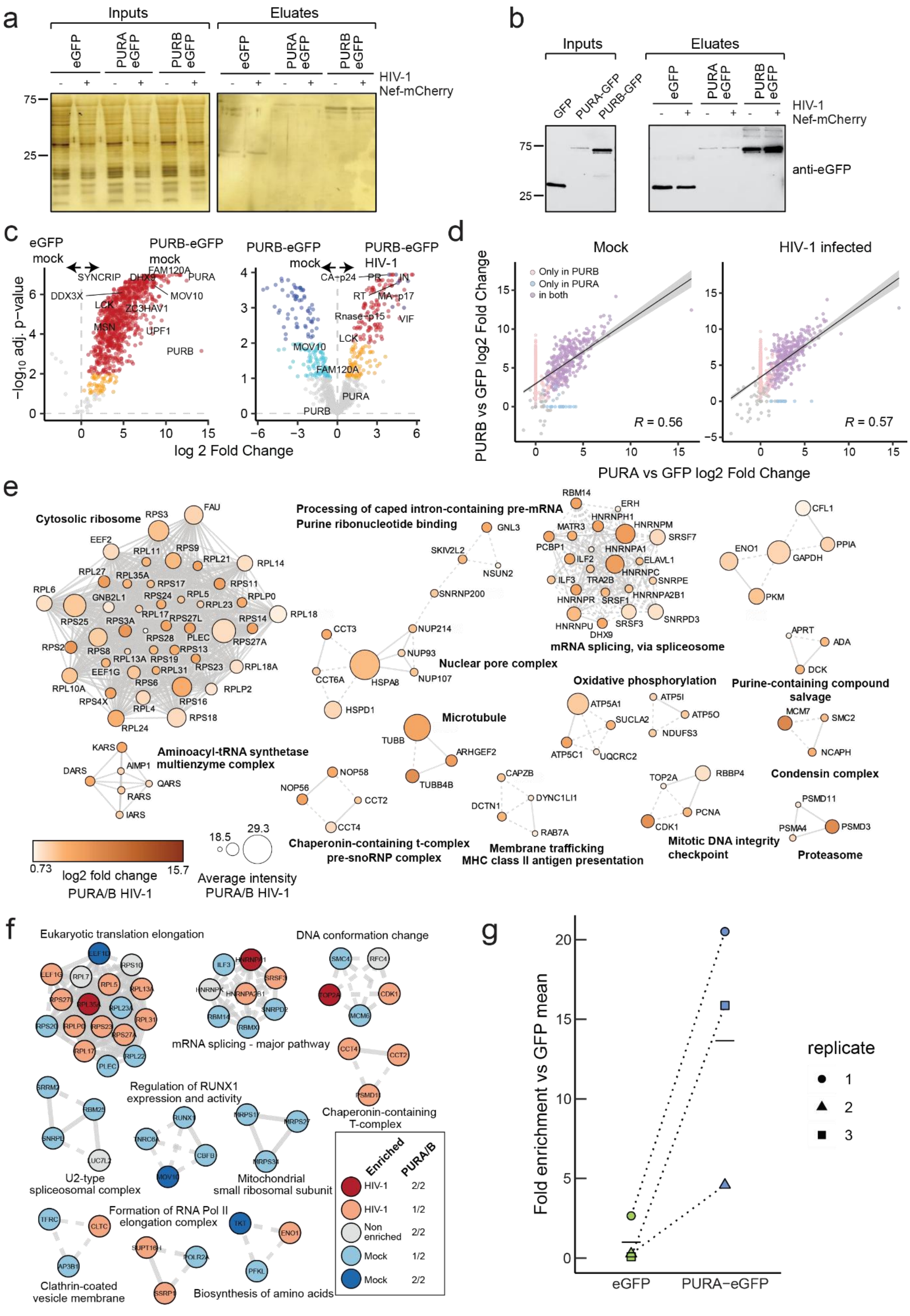

a
Inputs
Eluates
eGFP
PURA eGFP
PURB eGFP
HIV-1 Nef-mCherry
75
25
b
Inputs
Eluates
GFP
PURA-GFP
PURB-GFP
anti-eGFP
c
eGFP mock
PURB-eGFP mock
PURB-eGFP HIV-1
−log10 adj. p-value
log 2 Fold Change
SYNCRIP
DHX9
FAM120A
PURA
DDX3X
MOV10
LCK
ZC3HAV1
MSN
UPF1
PURB
CA−p24
PR
IN
RT
MA−p17
Rnase−p15
VIF
FAM120A
d
Mock
HIV-1 infected
Only in PURB
Only in PURA
in both
PURB vs GFP log2 Fold Change
PURA vs GFP log2 Fold Change
R = 0.56
R = 0.57
e
Cytosolic ribosome
Processing of caped intron-containing pre-mRNA
Purine ribonucleotide binding
mRNA splicing, via spliceosome
Nuclear pore complex
Oxidative phosphorylation
Purine-containing compound salvage
Microtubule
Condensin complex
Aminoacyl-tRNA synthetase multienzyme complex
Chaperonin-containing t-complex
pre-snoRNP complex
Membrane trafficking
MHC class II antigen presentation
Mitotic DNA integrity checkpoint
Proteasome
log2 fold change PURA/B HIV-1
Average intensity PURA/B HIV-1
f
Eukaryotic translation elongation
DNA conformation change
mRNA splicing - major pathway
Regulation of RUNX1 expression and activity
Chaperonin-containing T-complex
U2-type spliceosomal complex
Mitochondrial small ribosomal subunit
Formation of RNA Pol II elongation complex
Clathrin-coated vesicle membrane
Biosynthesis of amino acids
Enriched
PURA/B
HIV-1
Non enriched
Mock
g
Fold enrichment vs GFP mean
eGFP
PURA−eGFP
replicate
1
2
3

Supplemental Figure 8 (Figure S8): The protein interactome of PURA and PURB in HIV-1-infected cells. a-b) Silver staining (a) and Western blot with antibodies against eGFP (b) showing inputs and eluates of the IPs with the eGFP nanobody (GFP_Trap). c) Volcano plots showing the enrichment of the PURB-eGFP IP over the eGFP IP (left panel) and PURB-eGFP IP in HIV-1-infected over mock cells (right panel). Red and dark blue dots are proteins enriched with 1% FDR, while orange and cyan dots are proteins enriched with 10% FDR. Grey dots are non-enriched proteins. d) Scatter plot showing the correlation between PURA-eGFP and PURB-eGFP co-precipitated proteins. e) Cytoscape analysis of proteins differentially associated to PURA-eGFP. Top GO terms for each complex are shown. Solid lines represent physical interactions and dashed lines functional interactions. f) As in (e) but for proteins differentially associated to PURA-eGFP or/and PURB-eGFP in mock and HIV-1 infected cells. g) UV crosslinking and immunoprecipitation of eGFP or PURA-eGFP from HIV-1R-E-Nef-mCherry particles assembled in inducible HEK293 Flp-In T-Rex cells. HIV-1 gRNA was detected by RT-qPCR with viral specific primers. Lack of statistical significance is due to differences in dynamic range between experiments, but the trend is maintained across replicates. Related to Figure 4.

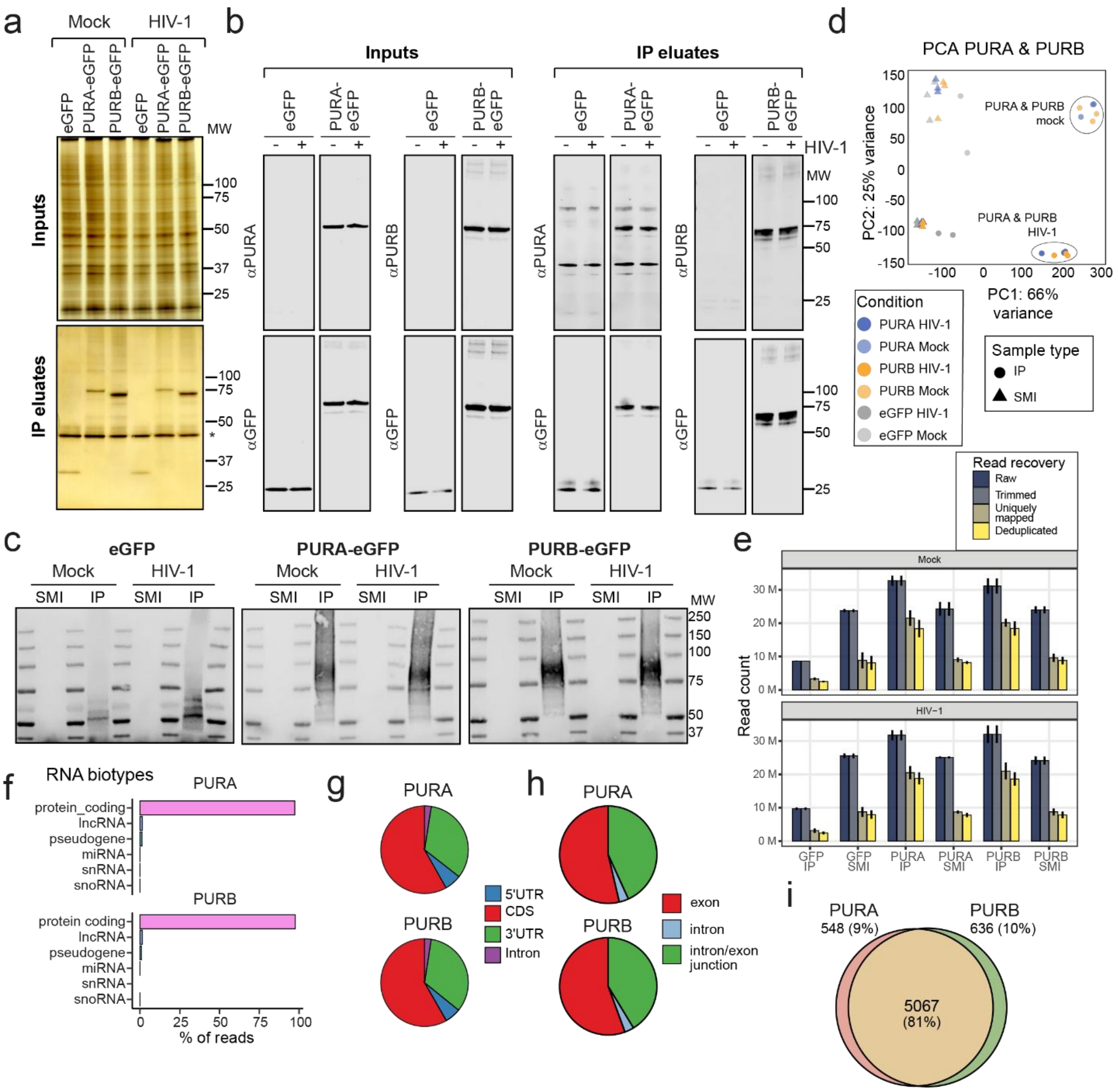

Supplemental Figure 9 (Figure S9): Analysis of PURA and PURB RNA targets. a-b) Silver staining (a) and Western blot (b) of the inputs and eluates of the IPs with eGFP_Trap for the iCLIP2 experiment. c) Analysis of the RNA co-purified with the immunoprecipitated proteins by ligation of a fluorescent DNA linker at the 3’ end and separation by SDS-PAGE. d) PCA of the different iCLIP2 samples. e) Quality control analysis of iCLIP2 reads. f) Bar plot showing the RNA biotypes co-purified with PURA-eGFP and PURB-eGFP. g) Pie chart showing the distribution of the PURA-eGFP and PURB-eGFP binding sites across mRNA features. h) as in (g) but within exons, introns and spanning intron/exon junctions. i) Venn diagram showing the overlapping between PURA-eGFP and PURB-eGFP bound transcripts. Related to Figure 5.

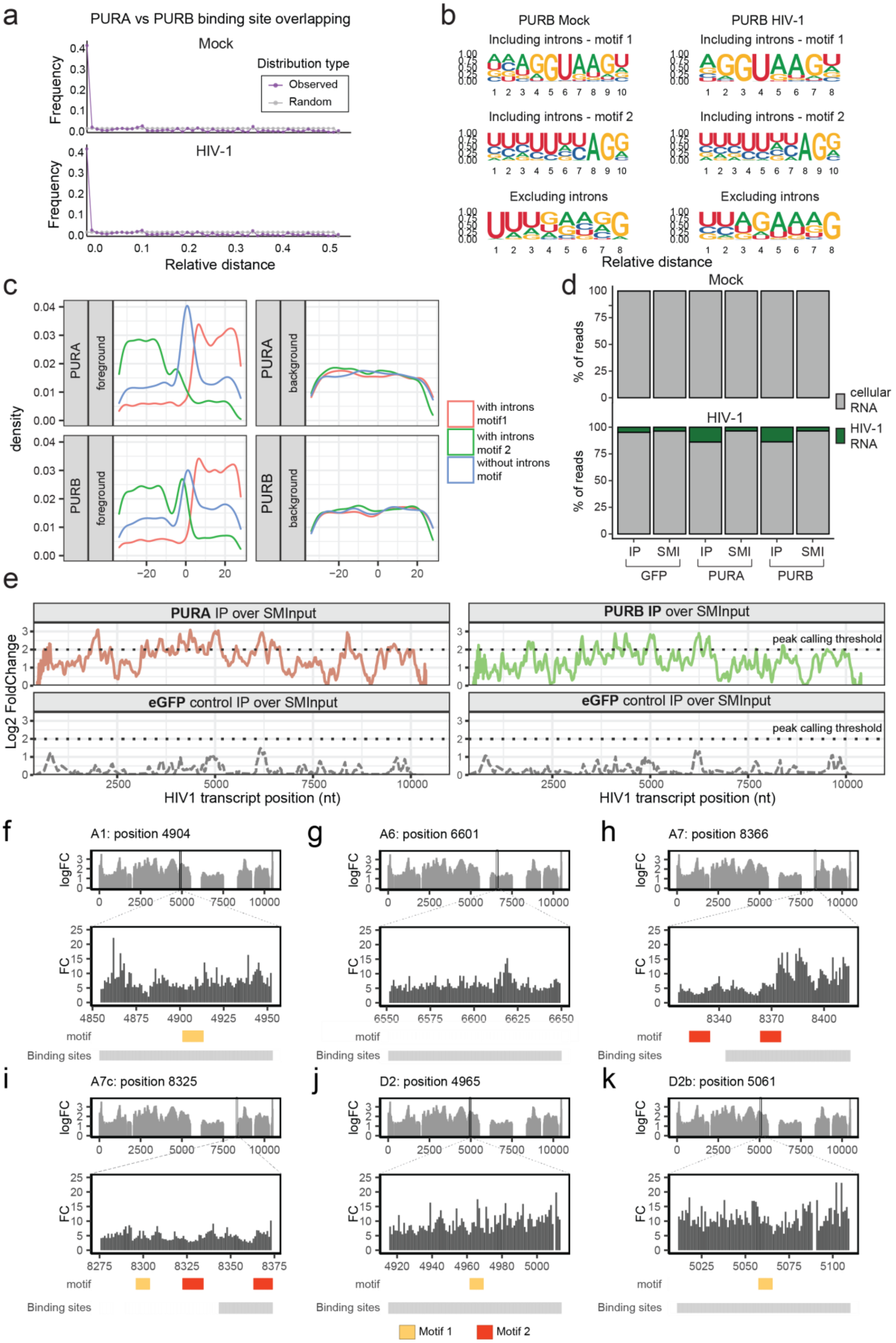

a
PURA vs PURB binding site overlapping
Mock
HIV-1
Distribution type
Observed
Random
Frequency
Relative distance
b
PURB Mock
PURB HIV-1
Including introns - motif 1
Including introns - motif 2
Excluding introns
Relative distance
c
PURA
PURB
foreground
background
density
with introns motif1
with introns motif 2
without introns motif
d
Mock
HIV-1
% of reads
cellular RNA
HIV-1 RNA
IP
SMI
GFP
PURA
PURB
e
PURA IP over SMInput
PURB IP over SMInput
eGFP control IP over SMInput
peak calling threshold
Log2 FoldChange
HIV1 transcript position (nt)
f
A1: position 4904
g
A6: position 6601
h
A7: position 8366
i
A7c: position 8325
j
D2: position 4965
k
D2b: position 5061
logFC
FC
motif
Binding sites
Motif 1
Motif 2

Supplemental Figure 10 (Figure S10): Analysis of PURA and PURB binding specificity on cellular and HIV-1 gRNA. a) Plot showing the relative distance between PURA-eGFP and PURB-eGFP binding sites. b) Analysis of the sequence motifs recognised by PURB-eGFP using the motif discovery software HOMER and including or excluding introns. c) Density plot showing the distribution of the sequence motifs across the binding site for PURA and PURB. d) Proportion of iCLIP2 reads mapping to human or HIV-1 genome in infected and uninfected cells. e) Binding site profile of PURA-eGFP and PURB-eGFP over SMI on the HIV-1 RNA genome, compared to that of unfused eGFP. f-k) Distribution of PURA binding sites across exon-intron junctions in the HIV-1 genome indicating the presence of matching binding motifs. Related to Figure 5.
